# Engineered Lipid Nanoparticles with Promoted Endosomal Escape and R283S-Mediated Stimulator of Interferon Genes (STING) Activation for Pancreatic Cancer Immunotherapy

**DOI:** 10.3390/pharmaceutics18060760

**Published:** 2026-06-21

**Authors:** Sizhen Wang, Qiwei Tai, Kehui Wang, Jianyu Zheng, Beibei Guo, Feng Yang, Chen Wang

**Affiliations:** 1 School of Pharmacy, Naval Medical University, Shanghai 200433, China; wsz08242546@163.com (S.W.); m13764705533_1@163.com (Q.T.); piu630qwq@163.com (K.W.); zhengjianyu2024@163.com (J.Z.); bbguo1994@163.com (B.G.); 2 Department of Oncology, Xinhua Hospital, School of Medicine, Shanghai Jiao Tong University, Shanghai 200092, China

**Keywords:** lipid nanoparticle, mRNA therapy, endosomal escape, STING activation, pancreatic cancer

## Abstract

**Background/Objectives**: Lipid nanoparticles (LNPs) have emerged as crucial vehicles for messenger RNA (mRNA) applications in antitumor therapy. Combining LNPs with stimulator of interferon genes (STING) activation holds promise for treating “cold” tumors such as pancreatic cancer. However, two major challenges remain: inefficient mRNA escape from endosomes and STING pathway suppression in immunosuppressive tumor microenvironments. **Methods**: To improve endosomal escape, we developed a novel pH-responsive PEGylated lipid (Ben-mPEG_2000_) for mRNA-LNP preparation while using commercial Man-mPEG_2000_ for dendritic cell (DC)-targeted delivery of LNPs; to alleviate suppression of the STING pathway in the tumor microenvironment and activate immune responses, STING-R283S mRNA was encapsulated into LNPs, ultimately resulting in DC-targeted/pH-responsive LNPs loaded with STING-R283S mRNA for pancreatic cancer immunotherapy research. **Results**: After pH-responsive cleavage, Ben-mPEG_2000_ not only enhanced the positive charge of LNPs through the exposed protonated amino groups but also eliminated the PEG-induced steric hindrance effect. The combination of these two effects promoted membrane fusion between LNPs and the endosome, thereby enhancing mRNA translation. As a payload, STING-R283S could further amplify STING signaling in DCs without cytotoxicity to counteract immunosuppression in pancreatic cancer. **Conclusions**: This engineered LNP platform enhanced mRNA expression and STING activation in DCs, improving immunotherapy outcomes in pancreatic cancer.

## 1. Introduction

Pancreatic cancer is a highly aggressive malignancy of the digestive tract with a median 5-year survival rate of merely 12–13% [1]. Consequently, over 80% of patients present with locally advanced or metastatic disease at initial diagnosis, rendering them ineligible for curative resection [2]. Currently, first-line systemic therapy for resectable or metastatic pancreatic cancer relies on gemcitabine-based regimens—either as monotherapy or in combination; however, these approaches yield only modest survival benefits and are frequently limited by hematologic and non-hematologic toxicities [3]. Precision-targeted therapeutics such as erlotinib and gefitinib, which selectively inhibit the epidermal growth factor receptor (EGFR), have demonstrated minimal clinical impact, largely attributable to primary and acquired resistance mechanisms [4]. Immune checkpoint inhibitors (ICIs) targeting PD-1/PD-L1 or CTLA-4 have revolutionized the treatment of immunologically “hot” tumors; however, they exhibit negligible efficacy in pancreatic cancer. More than 98% of patients with pancreatic cancer exhibit primary resistance to PD-1/PD-L1 blockade monotherapy [5,6], underscoring the inadequacy of single-agent ICIs in this setting. This resistance stems from a profoundly immunosuppressive tumor microenvironment (TME), characterized by sparse CD8^+^ T-cell infiltration and intrinsically low tumor mutational burden and neoantigen expression [7,8,9,10,11]. Collectively, these features result in poor antigen presentation (dendritic cells [DCs] act as the main executors) and impaired T-cell priming and trafficking, thereby undermining ICI activity. Importantly, robust intratumoral infiltration and functional persistence of DCs and CD8^+^ T cells—not merely their presence—are strongly correlated with improved progression-free and overall survival in patients with pancreatic cancer [12,13]. Therefore, achieving sustained, spatially coordinated infiltration and activation of DCs and antigen-specific CD8^+^ T cells within the pancreatic TME represents a pivotal therapeutic objective and a prerequisite for unlocking the full potential of immunotherapy in this recalcitrant malignancy.

Stimulator of interferon genes (STING) is an important regulatory factor in the natural immune response and antitumor response, and its signaling pathway can induce the secretion of type I interferons (IFN-I) and a series of cytokines, including CXCL10 and CCL5 [14,15,16,17]. IFN-I can inhibit tumor growth directly and indirectly by acting upon tumor and immune cells [18]. For instance, STING activation promotes the differentiation and adhesive capacity of immune cells (including dendritic cells) and supports the expansion of antitumor T lymphocytes after their infiltration into tumor sites [19,20,21,22]. Concurrently, chemokines such as CXCL10 and CCL5 play pivotal roles in guiding effector T cells with tumor specificity into the tumor bed [23,24,25]. Collectively, triggering the STING pathway within the TME can effectively reprogram an immunologically inert (“cold”) TME into an inflamed, T-cell-infiltrated (“hot”) state conducive to antitumor immunity [26,27,28,29,30]. Moreover, IFN-I and a series of cytokines produced upon STING pathway activation (e.g., CXCL-10, TNF-α) can induce lethal DNA damage and direct tumor cell death, thereby acting as damage-associated molecular patterns (DAMPs) to stimulate the immune response [18,31,32]. However, traditional STING agonists can induce T-cell cytotoxicity, counteracting the desired antitumor immune response [33,34,35]. Importantly, research has found that STING-R283S mRNA (the human homologous mutation is denoted as R284S) does not induce T-cell cytotoxicity, thereby reactivating the antitumor response without exerting antiproliferative effects on lymphocytic immune cells, thus overcoming the toxicity and limitations of conventional STING agonists [35,36,37]. Additionally, STING-R283S is a mutant that can spontaneously aggregate and continuously activate downstream signaling pathways without upstream signals (such as 2′,3′-cGAMP), which is beneficial for treating pancreatic tumors with low expression of 2′,3′-cGAMP or cGAS [35]. Therefore, using STING-R283S mRNA instead of STING agonists to achieve attenuation and enhancement has great research value for antitumor immunotherapy. Delivering the nucleic acid molecules encoding the STING-R283S sequence to DCs while initiating the antitumor immune response overcomes the toxic side effects of traditional STING agonists and is expected to become an effective treatment for pancreatic cancer.

Lipid nanoparticle (LNP)-based mRNA technology has recently pushed tumor immunotherapy to the forefront of oncology research [38,39,40]. However, endosomal escape is an important bottleneck faced by mRNA-LNP technology [41,42,43,44]. Studies have shown that approximately 70% of mRNA cannot achieve endosomal escape; some mRNAs will be degraded by lysosomes, while others will be expelled by cells due to the inability to undergo timely membrane fusion during endosomal circulation [45,46]. Therefore, improving the endosomal escape ability of mRNA is essential for promoting mRNA expression and stimulating efficient immune responses. In addition, regarding the issue of effective expression of mRNA, some studies have shown that a novel ionizable lipid based on N1, N3, N5-tris(2-aminoethyl)benzene-1,3,5-tricarboxamide (BXA) has extremely high transfection efficiency and can effectively encapsulate and deliver various types of mRNA [47,48]. In addition, as an auxiliary lipid material, PEGylated lipids are an indispensable component in LNPs; they can reduce the particle size of LNPs, improve their stability, and avoid sedimentation and aggregation phenomena. Additionally, PEG modification on the surface of LNPs can form a fixed water layer structure, thereby resisting the adsorption of serum proteins through electrostatic and hydrophobic interactions and interfering with the uptake of the reticuloendothelial system (RES) through spatial steric hindrance effects [49]. However, the space hindrance and hydrophilic effect caused by excessive PEGylated lipid modification not only affect the uptake of cells and the internalization of LNPs but also interfere with endosomal membrane fusion between LNPs and DCs, hindering the release of mRNA and affecting its expression function in the cytoplasm, thereby reducing the therapeutic efficacy and causing adverse reactions [50,51,52].

**Figure 1:**
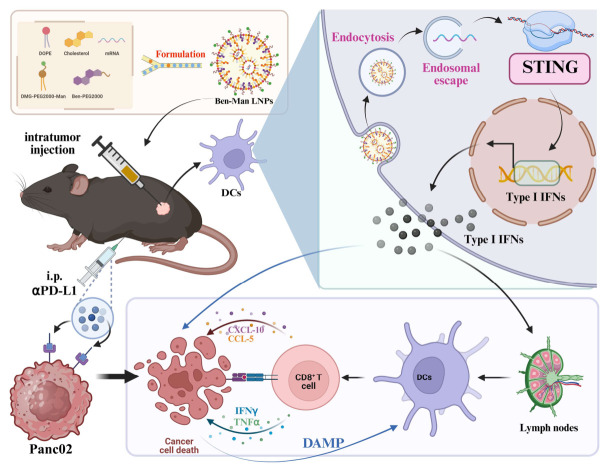
Schematic illustration of the fabrication and immunotherapy mechanism of Ben-Man LNP/STING-R283S mRNA in pancreatic cancer. (Created in BioRender. Weiwei, J. (2026) https://BioRender.com/lhsl7hz (accessed on 5 June 2026)) DCs: dendritic cells; i.p.: intraperitoneal injection; Type I IFNs: type I interferons; STING: stimulator of interferon genes; DAMP: damage-associated molecular patterns; IFNγ: interferon-gamma; TNFα: tumor necrosis factor-alpha.

To overcome the aforementioned problems, we designed a pH-responsive PEGylated lipid (Ben-mPEG_2000_) based on a benzamide bond. The modification of Ben-mPEG_2000_ can further reduce the particle size of LNPs, making it easier for cells to absorb and internalize them. Ben-mPEG_2000_ can break the chain and detach from the surface of LNPs in the acidic environment of the endosome (pH 5.0–6.5), reducing the spatial obstruction of LNPs, decreasing the hydrophilic effect, and simultaneously exposing free primary amines and undergoing protonation, promoting the fusion of LNPs with the endosomal membrane, assisting mRNA endosomal escape and release, and thereby enhancing the expression efficiency of mRNA. Meanwhile, commercialized mannose-modified PEGylated lipids were used for modification to achieve targeted delivery of DCs, and BXA ionizable lipid was used to further enhance the transfection efficiency of mRNA-LNP. The DC-targeting/pH-responsive LNP platform (Ben-Man LNP) was used to deliver STING-R283S mRNA, a novel immunomodulatory strategy that does not rely on upstream STING activation factors, for pancreatic cancer immunotherapy. We demonstrated that Ben-Man LNPs can selectively deliver mRNA to DCs, assisting in the powerful endosomal escape function and triggering strong and sustained activation of the STING signaling pathway, thereby leading to strong IFN production and DC maturation, while the released IFNs promote DNA damage in tumor cells following DAMP release, enhancing cross-stimulation of tumor-specific CD8^+^ T cells. Therefore, this method effectively matures DCs and promotes the accumulation of functional CD8^+^ T cells within the tumor, thereby triggering a systemic and persistent antitumor immune response. Additionally, when combined with ICIs and co-stimulatory agonists, mRNA-LNP can overcome several key obstacles in antitumor immunity, including poor antigen presentation and T-cell rejection (Figure 1). In summary, this study lays a translational foundation for the application of STING mRNA therapeutic approaches targeting DCs in pancreatic cancer and emphasizes the significant advantages of STING-R283S as an immune therapy mode that is not antigen-dependent, especially in pancreatic cancer, a disease with a very low burden of tumor-specific antigens, where conventional vaccines and T-cell receptor-based methods face fundamental limitations.

## 2. Materials and Methods

### 2.1. Materials

The ionizable lipids DLin-MC3-DMA, 1,2-dioctadecanoyl-sn-glycero-3-phosphocholine (DSPC, #S01005), and cholesterol (Chol, #O01001) were purchased from AVT Pharmaceutical Tech Co., Ltd. (Shanghai, China). 1,2-Dioleoyl-sn-glycero-3-phosphoethanolamine (DOPE, #768593), DMG-mPEG_2000_-Man (N/A) and Cy5-cholesterol (N/A) were obtained from Xi’an Ruixi Biological Technology Co., Ltd. (Xi’an, China); mCherry mRNA (N/A) and Cy5-mCherry mRNA (N/A) were obtained from Humantech Co., Ltd. (Shanghai, China); and the STING-R283S mRNA (N/A) was customized by GENEWIZ Co., Ltd. (Suzhou, China). We synthesized the ionizable lipid BXA2-8 and pH-responsive benzamide-based mPEG_2000_ lipid (Ben-mPEG_2000_) in our laboratory (procedure available in Supplementary Materials). p-STING (#72971), TBK1 (#3504), p-TBK1 (#5483), IRF3 (#4302), p-IRF3 (#29047), and MHC-I (#76828) mAbs were purchased from Cell Signaling Technology (Danvers, MA, USA). STING primary antibodies (#ab181125) and anti-rabbit IgG H&L (HRP, #ab6721) were obtained from Abcam (Boston, MA, USA), and anti-PD-L1 mAb (#BE0101) was purchased from the BioXcell Company (West Lebanon, NH, USA).

### 2.2. mRNA-Encapsulated LNP Preparation

mRNA-loaded LNPs were fabricated via microfluidic mixing under controlled conditions. The optimized molar composition of Ben-Man LNPs was BXA2-8:cholesterol:DOPE:DMG-mPEG2000-Man:Ben-mPEG_2000_ = 30:40:30:0.375:1.125. In contrast, the reference formulations—BXA LNPs and BXA-Man LNPs—shared identical base lipid ratios (BXA2-8:cholesterol:DOPE:DMG-mPEG_2000_ = 30:40:30:0.375), differing only in the presence or absence of the mannose-conjugated PEG lipid (i.e., DMG-mPEG_2000_-Man). As a benchmark, commercially derived MC3-based LNPs were prepared with the standard formulation DLin-MC3-DMA:cholesterol:DSPC:DMG-mPEG2000 = 50:38.5:10:1.5. For fluorescence tracking, Cy5-labeled mCherry mRNA was incorporated into LNPs for in vitro studies; for in vivo cellular uptake experiments, 1.5 mol% of cholesterol was replaced with an equimolar amount of Cy5-cholesterol. Immediately after synthesis, freshly formed LNPs were diluted 10-fold with ice-cold 1× PBS, followed by concentration using Amicon Ultra-15 centrifugal filters (100 kDa MWCO; MilliporeSigma, St. Louis, MO, USA). The resulting purified, mRNA-encapsulated LNP suspensions were stored at 4 °C for subsequent analysis.

### 2.3. Characterization

Physicochemical characterization of the LNPs was performed as follows: hydrodynamic diameter and surface charge (zeta potential) were determined by dynamic light scattering (DLS) using a Malvern Zetasizer Nano ZSE (Malvern Panalytical, Malvern, UK). Structural morphology was visualized by cryo-transmission electron microscopy (cryo-TEM) on a Tecnai G2 F20 S-Twin microscope (FEI Company, Hillsborough, OR, USA) operating at 200 kV. To quantify mRNA encapsulation, the QuantiFluor^®^ RNA System (Thermo Fisher Scientific, Waltham, MA, USA) was employed—replacing the older RiboGreen assay with an updated, more robust fluorometric method. Encapsulation efficiency (EE%) was calculated as follows:

EE% = [(Total RNA concentration − Free RNA concentration)/Total RNA concentration] × 100%,

where “Total RNA concentration” refers to the overall mRNA content in the LNP formulation, and “Free RNA concentration” denotes the amount of non-encapsulated (supernatant-associated) mRNA remaining after separation via ultrafiltration.

### 2.4. Measurement of Ben-mPEG_2000_ Modification Efficiency

The Ben-mPEG_2000_ modification efficiency was quantified by detecting the remaining primary amine groups using 2,4,6-trinitrobenzenesulfonic acid (TNBS) [46]. Briefly, 100 µL of the LNP solution was mixed with borate buffer (200 µL, 0.1 M, pH 9.5) and Triton X-100 (10% *w*/*v*, 50 µL). After 20 min incubation at room temperature, 100 µL of TNBS (0.1% *v*/*v*) was added, and the solution was further incubated in the dark at room temperature for 1 h; the absorbance was then measured at 420 nm and normalized against Compound 2. The modification efficiency (ME) was calculated as follows:

ME (%) = 1 − (A_U_ − A_U0_)/(A_T_ − A_T0_) × 100%

where A_U_ is the absorbance of the Ben LNP solution, A_U0_ is the blank solvent absorbance, A_T_ is the absorbance of Compound 2, and A_T0_ is the corresponding blank solvent.

### 2.5. Assessment of Ben-mPEG_2000_ Hydrolysis Under Physiologically Relevant pH Conditions

Primary amine generation resulting from Ben-mPEG_2000_ hydrolysis was quantified using the TNBS assay [47]. Briefly, 0.003 mmol of pH-responsive Ben-mPEG_2000_ was dissolved in 1 mL of PBS buffer at each of the specified pH values (pH 5.5, 6.5, and 7.4). The solution was incubated at 37 °C with shaking (200 rpm), and 100 μL was withdrawn at predetermined time points. Immediately after sampling, the same volume of pre-warmed PBS buffer was replenished. Next, 100 μL of the withdrawn solution was transferred to 1 mL of borate buffer (pH 10.0), followed by the addition of 100 μL of TNBS solution (0.1% *v*/*v*), and the mixture was thoroughly mixed. The resulting mixture was incubated at 50 °C for 1 h. The absorbance was measured at 420 nm using a microplate reader. PBS buffers at pH 5.5, 6.5, and 7.4 were used as blank controls, and Compound 2 was used as the positive control. The hydrolysis rate (HR) was calculated as follows:

HR% = (A_sample_ − A_blank_solvent_)/(A_reference_ − A_blank_reference_) × 100%,

where A_sample_ denotes the absorbance of hydrolyzed Ben-mPEG_2000_ at a given time point, A_blank_solvent_ is the absorbance of the solvent control, A_reference_ corresponds to the absorbance of Compound 2, and A_blank_reference_ represents the blank absorbance measured for Compound 2.

### 2.6. Isolation of BMDCs and SLCs

Bone marrow-derived dendritic cells (BMDCs) and splenic lymphocytes (SLCs) were extracted from 7-week-old C57BL/6 mice, and cultivation was performed according to relevant references [47] and the procedure available in Supplementary Materials.

### 2.7. Cellular Uptake In Vitro

DC2.4 cells (Vigen Biotechnology Co., Ltd., Zhenjiang, China; catalog #VGC-0534-0000) were plated at a density of 1 × 10^5^ cells per well in complete culture medium and allowed to adhere for 12 h. Cells were then treated with Cy5-labeled mCherry-encoding LNPs containing 600 ng/mL mRNA for 1 or 4 h. After incubation, nuclei were counterstained with Hoechst 33342 (5 µg/mL) at 37 °C for 10 min. Unbound dye was removed by three gentle washes with pre-warmed PBS (pH 7.4). Subcellular localization was visualized using confocal laser scanning microscopy (CLSM, Zeiss LSM 710, Carl Zeiss, Jena, Germany). Imaging parameters were set as follows: Cy5 was excited at 649 nm and detected at 670 nm; Hoechst 33342 was excited at 405 nm and emitted at 488 nm.

### 2.8. In Vitro Assessment of Cellular Viability

Cytotoxicity of Ben-Man LNPs loaded with mRNA was evaluated in DC2.4 cells (seeded at 1 × 10^4^ cells/well in flat-bottom 96-well plates) and BMDCs (seeded at 1 × 10^4^ cells/well in U-bottom 96-well plates). After a 12 h adherence period, cells were exposed to Cy5-labeled mCherry-encoding LNPs across a range of concentrations for 24 h. Viability was then determined using the Cell Counting Kit-8 (CCK-8; Dojindo Laboratories, Kumamoto, Kumamoto Prefecture, Japan).

### 2.9. In Vitro Evaluation of DC-Specific Delivery and mRNA Transfection Efficiency

DC2.4 cells (1 × 10^5^ cells per well) and BMDCs (5 × 10^5^ cells per well) were incubated with LNPs encapsulating mCherry-encoding mRNA (600 ng/mL) for 24 h. Fluorescence signal from expressed mCherry was quantified using a multimode microplate reader (SpectraMax i3x, Molecular Devices, Sunnyvale, CA, USA) with excitation at 590 nm and emission detection at 645 nm.

### 2.10. Evaluation of Endosomal Escape Using Calcein Leakage Assay

The calcein leakage assay was conducted following a previously established protocol [47]. DC2.4 cells were seeded and rinsed with phosphate-buffered saline prior to incubation with fresh culture medium supplemented with 100 μM calcein and mRNA-loaded LNPs (600 ng/mL) for 2 h. Subsequently, cells were processed for CLSM analysis.

### 2.11. STING Pathway Activation by Western Blot In Vitro

DC2.4 cells (1 × 10^6^ cells per well) were exposed to LNPs encapsulating STING-R283S mRNA (600 ng/mL) for 4 h. Whole-cell lysates were prepared, and proteins were resolved by SDS-PAGE before being electrotransferred onto PVDF membranes. After blocking, membranes were probed overnight at 4 °C with primary antibodies (1:1000) targeting STING, TBK1, IRF3, and their phosphorylated forms (p-STING, p-TBK1, p-IRF3). HRP-conjugated secondary antibodies (1:5000) were applied subsequently, and immunoreactive bands were visualized using enhanced chemiluminescence (ECL) detection.

### 2.12. In Vitro Assessment of Immune Cell Activation

BMDCs transfected with STING-R283S mRNA-loaded LNPs (600 ng/mL) for 12 h were subsequently co-cultured with SLCs and Panc02 tumor cells at a ratio of 1:5:1 for an additional 12 h. Culture supernatants were harvested to measure concentrations of key immunomodulatory cytokines, including IFN-β, IL-6, IL-12, CXCL-10, IFN-γ, and TNF-α, using commercial ELISA kits. Parallel samples were subjected to flow cytometric analysis to characterize DC activation status (CD11c^+^, CD80^+^ CD86^+^) and T-cell composition (CD3^+^ CD4^+^, CD3^+^ CD8^+^) with flow antibodies diluted 100 times. The group that was not treated with LNPs was the control group.

### 2.13. In Vitro Evaluation of Specific Cytolytic Activity

Cytotoxic T lymphocyte (CTL)-mediated killing capacity was assessed via the CCK-8 assay and lactate dehydrogenase (LDH) assays. Panc02 cells (Vigen Biotechnology Co., Ltd., Zhenjiang, China, #VGC-0540-0000, 1 × 10^4^ cells/well) were seeded in 96-well plates. BMDCs (1 × 10^4^ cells/well) transfected with STING-R283S mRNA-encapsulated LNPs (600 ng mRNA/mL) for 12 h were co-cultured with Panc02 and SLCs (Panc02:BMDCs:SLCs = 1:1:5) for another 24 or 48 h. Supernatants were analyzed for LDH release, and the viability of the remaining attached Panc02 cells was assessed using the CCK-8 kit. The group that was not treated with LNPs was the free cell group, and the Pan02 cells cultured only in the medium were used as the control group.

### 2.14. Treatment of Panc02 Tumors In Vivo

A total of 77 male C57BL/6 mice (6 weeks old) were used in the therapeutic study. Each mouse received a subcutaneous injection of 1 × 10^6^ Panc02 tumor cells. All animal procedures were conducted in accordance with institutional guidelines and approved by the Ethics Committee of Medicine, Naval Medical University (approval number: NMUMREC-2024-82473891). Mice were randomly assigned to seven experimental groups (*n* = 11 per group): Ben-Man+αPD-L1, Ben-Man, Man, BXA, αPD-L1, MC3, and PBS. Among them, five were used for pharmacodynamic studies, such as hematoxylin and eosin (H&E), ELISA and so on, and six were used for survival analysis. Treatment commenced once subcutaneous tumors reached a volume of 80–100 mm^3^. The entire treatment cycle lasted 20 days, starting from the administration of the medication. Mice were administered either PBS or 10 μg of STING-R283S mRNA formulated in LNPs via intratumoral injection on days 0, 5, 10, and 15. In combination therapy groups, mice additionally received intraperitoneal injections of 100 μg anti-PD-L1 monoclonal antibody. Tumor dimensions were monitored regularly throughout the study. On day 20, mice were humanely euthanized, and tumor tissues and blood were harvested for downstream analyses, including flow cytometry, ELISA, H&E staining, immunofluorescence imaging, serum biochemical profiling, and bulk RNA sequencing.

### 2.15. Intratumoral Cell Uptake of mRNA-LNP

Twelve male C57BL/6 mice (6 weeks old) were subcutaneously implanted with 1 × 10^6^ Panc02 cells. The mice were randomly divided into four groups (*n* = 3): Ben-Man, Man, BXA, and MC3. Upon reaching 80–100 mm^3^, intratumoral injection of Cy5-LNP was initiated. After 12 h of intratumoral administration, the mice were euthanized by CO_2_ asphyxiation using a gradual-fill method, and the tumor tissues were removed. The co-localization of CD11c^+^ DCs and Cy5-LNP was analyzed using frozen sections combined with immunofluorescence technology, and the co-localization coefficient was analyzed using ImageJ (v1.8.0)-JACoP.

### 2.16. Flow Cytometry Analysis and ELISA Assay In Vivo

On the 17th day after administration, the mice were euthanized by CO_2_ asphyxiation using a gradual-fill method, and the tumor tissues were extracted. Tumor tissues were mechanically dissociated and enzymatically digested to generate single-cell suspensions, which were then subjected to flow cytometric analysis on a Sony spectral flow cytometer to quantify immune cell subsets and assess surface marker expression by staining with fluorochrome-conjugated antibodies against CD11c, CD80, CD86, MHC-II, CD45, CD3, CD4, and CD8a (Thermo Fisher Scientific). For cytokine profiling, approximately 0.2 g of tumor tissue was homogenized in ice-cold RIPA lysis buffer containing protease inhibitors, followed by centrifugation to obtain clarified supernatants. Total protein concentration in each lysate was determined using a BCA assay. Levels of IFN-β, IFN-γ, TNF-α, and CXCL-10 were measured in the supernatants via commercial ELISA kits and normalized to both tissue weight and total protein content.

### 2.17. RNA Sequencing and MHC-I Detection

On the 20th day after administration, the mice in the Ben-Man LNP and BXA LNP groups were euthanized by CO_2_ asphyxiation using a gradual-fill method, and the tumor tissues were extracted for RNA sequencing analysis using the Illumina platform. To detect MHC-I protein expression, BMDCs (1 × 10^6^ cells/well) were treated with STING-R283S mRNA-encapsulated LNPs (600 ng mRNA/mL) and co-cultured with Panc02 cells (1 × 10^5^ cells/well) for 24 h. Supernatants from BMDC cultures were harvested, and whole-cell lysates were prepared. Proteins were resolved by SDS-PAGE and electroblotted onto PVDF membranes. After blocking, membranes were incubated overnight at 4 °C with anti-MHC-I primary antibodies (1:1000), followed by HRP-conjugated secondary antibodies (1:5000). Immunoreactive bands were detected using ECL.

### 2.18. Statistical Analysis

Statistical evaluations were conducted using GraphPad Prism (v10.3). Data are expressed as mean ± standard deviation (SD). Group-wise comparisons were performed using two-tailed unpaired Student’s *t*-test for pairwise analyses or one-way ANOVA with Tukey’s or Dunnett’s post hoc test for multi-group comparisons. Statistical significance was set at * *p* < 0.05, ** *p* < 0.01, *** *p* < 0.001, and **** *p* < 0.0001. The number of animals used is indicated in the corresponding figure legends.

## 3. Results

### 3.1. Ben-Man LNP Synthesis for mRNA Delivery

BXA ionizable lipid was synthesized according to a previous reference [47], and structural confirmation was carried out by high-resolution mass spectrometry (HRMS) (Figure S1) and nuclear magnetic resonance (^1^H NMR) (Figure S2). We successfully synthesized the Ben-mPEG_2000_ PEGylated lipid, following the representative synthetic pathways depicted in Figure S3. Its chemical structure was characterized using nuclear magnetic resonance (^1^H NMR) spectroscopy (Figure S4). The results were in close agreement with the theoretical chemical shifts and integral ratios of various hydrogen atoms in the Ben-mPEG_2000_ structure, indicating that Ben-mPEG_2000_ was successfully synthesized (Supplementary Materials).

**Figure 2:**
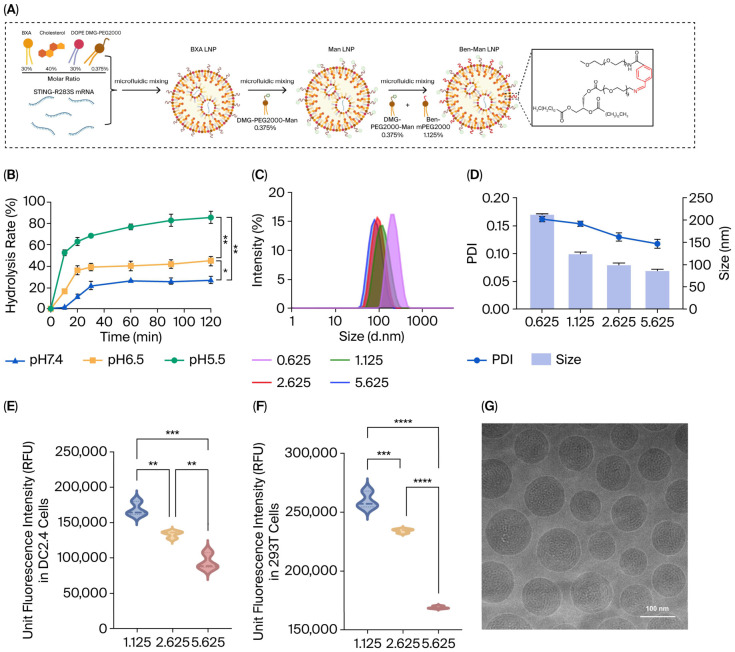
Synthesis and physicochemical characterization of Ben-Man–Based LNPs. (**A**) Schematic illustration of the microfluidic fabrication process for Ben-Man LNPs. Red: benzamide bond (Created in BioRender. Weiwei, J. (2026) https://BioRender.com/00tgh9f (accessed on 5 June 2026)) (**B**) pH-dependent hydrolytic degradation profile of Ben-mPEG2000, assessed across physiologically relevant pH conditions (n = 3). (**C**) Hydrodynamic diameter of Ben-Man LNPs formulated with varying molar percentages of Ben-mPEG_2000_: 0.625%, 1.125%, 2.625%, and 5.625% (relative to total lipid mass). (**D**) Size distribution profiles and polydispersity index (PDI) of the four Ben-Man LNP formulations, determined by dynamic light scattering (DLS). (**E**,**F**) In vitro transfection efficiency of mRNA-loaded Ben-Man LNPs in DC2.4 (**E**) and HEK293T (**F**) cells, comparing formulations containing 1.125%, 2.625%, or 5.625% Ben-mPEG2000 (n = 3 per group). (**G**) Cryo-transmission electron microscopy (cryo-TEM) images revealing the morphology and structural integrity of Ben-Man LNPs; scale bar = 100 nm. Statistical significance was evaluated using a one-way ANOVA with Tukey test (* *p* < 0.05; ** *p* < 0.01; *** *p* < 0.001; **** *p* < 0.0001). Data are presented as mean ± SD.

The initial BXA LNPs and Man LNPs were obtained by combining BXA2-8 ionizable lipid with cholesterol, DOPE, and DMG-mPEG_2000_ or DMG-mPEG_2000_-Man via microfluidic mixing. Subsequently, we incorporated a portion of Ben-mPEG_2000_ into the Man LNPs to synthesize Ben-Man LNPs (Figure 2A).

To evaluate the pH-responsive hydrolysis of Ben-mPEG_2000_, we quantified its degradation kinetics across physiologically relevant pH conditions. As shown in Figure 2B, the hydrolysis rate at pH 5.5 was significantly accelerated compared with that at pH 6.5 and 7.4 after 2 h of incubation (85.62% ± 5.73% vs. 44.85% ± 4.46% vs. 27.19% ± 3.14%, *p* < 0.05), demonstrating robust acid-triggered lability. Moreover, the benzamide bond exhibits pH-dependent hydrolysis: its cleavage rate increases progressively with decreasing pH. At physiological pH 7.4, hydrolysis remains limited, plateauing at approximately 30% over time; at acidic pH 5.5, which mimics endosomal environments, the bond undergoes significantly accelerated cleavage, reaching ~85% hydrolysis saturation and enabling efficient PEG shedding from the lipid conjugate.

A systematic screening strategy was implemented to identify the optimal molar percentage of Ben-mPEG_2000_ in Ben-Man LNPs, testing four formulations: 0.625%, 1.125%, 2.625%, and 5.625% (mol/mol relative to total lipid). LNPs formulated with 0.625% Ben-mPEG_2000_ exhibited a mean particle size exceeding 200 nm and poor size homogeneity (Figure 2C,D), rendering them unsuitable for downstream functional evaluation, and thus, they were excluded from further analysis. Grafting efficiency, quantified as the percentage of surface-exposed Ben-mPEG_2000_ successfully conjugated to the LNP surface, was assessed for the remaining three formulations (1.125%, 2.625%, 5.625%), and it is summarized in Figure S5. Among the three tested formulations (1.125%, 2.625%, 5.625%), the 1.125% Ben-mPEG_2000_ formulation (designated as Ben-Man LNP) exhibited the highest grafting efficiency on the LNP surface and demonstrated superior transfection efficiency in both DCs and 293T cells, and these findings informed our selection of the 1.125% formulation for subsequent experiments (Figure 2E,F).

The optimized Ben-Man LNP formulation exhibited a uniform spherical morphology, with a mean hydrodynamic diameter of 125.57 ± 3.30 nm (Figure 2G; Table S1). It achieved a high mRNA encapsulation efficiency of 96.49% ± 0.07% (Table S1), confirming robust payload retention under standard formulation conditions.

### 3.2. Ben-Man LNPs Demonstrated Enhanced Targeted Cellular Uptake Ability

**Figure 3:**
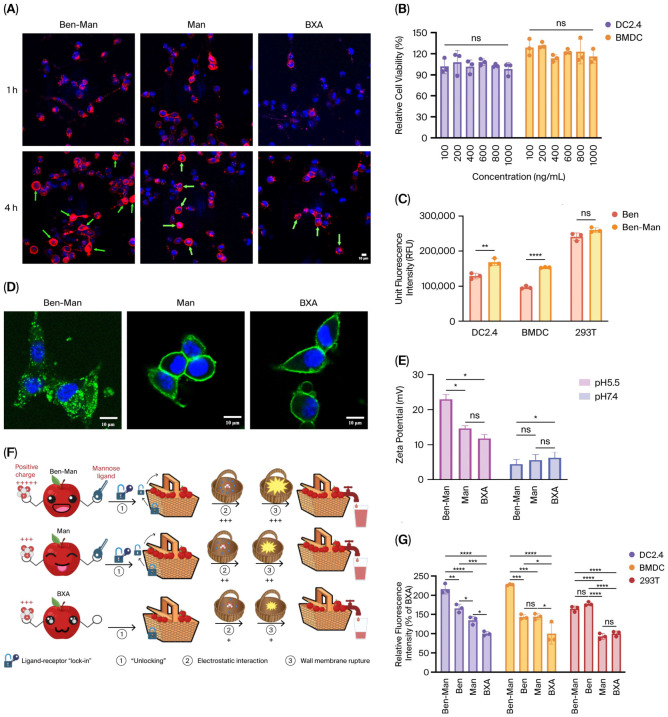
Cellular internalization, endosomal escape capacity, and mRNA delivery efficiency. (**A**) Time-dependent cellular uptake of Cy5-labeled mCherry mRNA delivered by distinct LNP formulations in DC2.4 cells, visualized by confocal microscopy at 1 h and 4 h post-incubation (red signal); nuclei counterstained with Hoechst 33342 (blue); scale bar = 10 µm. (**B**) Cytotoxicity assessment of Ben-Man LNPs in DC2.4 cells and BMDCs, quantified via cell viability assay (n = 3). (**C**) Comparative transfection efficiency of mannose-functionalized versus non-mannosylated LNPs carrying mCherry mRNA across DC2.4, BMDCs, and HEK293T cells. (**D**) Confocal imaging of calcein release (green) into the cytosol of DC2.4 cells following LNP treatment, indicating endosomal rupture; nuclei stained with Hoechst 33342 (blue); scale bar = 10 µm. (**E**) pH-responsive surface charge modulation: zeta potential measurements of various LNPs incubated at acidic (pH 5.5) versus physiological (pH 7.4) conditions. (**F**) Conceptual illustration depicting the dual-response mechanism of Ben-Man LNPs: mannose-mediated dendritic cell recognition coupled with pH-triggered endosomal destabilization to promote efficient cytosolic mRNA delivery (LNPs symbolized as apples; successful mRNA translation represented by apple juice). “+” means positive charge. (Created in BioRender. Weiwei, J. (2026) https://BioRender.com/nfgdfn3 (accessed on 5 June 2026)) (**G**) Quantitative transfection efficiency of mCherry mRNA-loaded LNPs in DC2.4, BMDCs, and HEK293T cells (n = 3 per group). Statistical significance was determined using one-way ANOVA with Tukey’s post hoc test or two-tailed unpaired *t*-tests where appropriate (* *p* < 0.05, ** *p* < 0.01, *** *p* < 0.001, **** *p* < 0.0001, ns: no significant difference); data are presented as mean ± SD.

The cellular uptake of Cy5-mCherry mRNA-encapsulated LNPs was assessed via confocal laser scanning microscopy. Both Ben-Man and Man LNPs demonstrated higher uptake in DCs than the BXA formulation (initial LNP) within 1 h (Figure 3A), corroborating the DC-targeting specificity conferred by surface-exposed mannose residues. Ben-Man LNPs achieved the highest uptake among all groups, attributable to two synergistic factors: mannose receptor-mediated recognition by DCs and size reduction induced by Ben-mPEG_2000_, which decreased the LNP hydrodynamic diameter and facilitated DC internalization. Consequently, accelerated endosomal escape and subsequent release of mCherry mRNA led to robust intracellular protein expression within 4 h, as evidenced by intense red fluorescence in the whole cell (green arrows).

Before in vitro transfection, we evaluated the cytotoxicity of mCherry mRNA expressed in Ben-Man LNPs in DC2.4 cells and BMDCs by quantifying cell viability after a 24 h exposure to a dose gradient of LNPs. Ben-Man LNPs exhibited excellent biocompatibility across all tested doses (>90%), including the highest mRNA concentration of 1000 ng/mL (Figure 3B).

To evaluate the DC-targeting capability of Ben-Man LNPs, we compared their mRNA transfection efficiency with that of unmodified glycosylated Ben LNPs in DC2.4 cells and BMDCs. Ben-Man LNPs achieved significantly higher transfection efficiency than Ben LNPs in both DC types (*p* < 0.01) (Figure 3C). This enhancement was attributable to the specific recognition of surface-expressed glycan receptors on DCs by the mannosylated ligands on Ben-Man LNPs, which facilitates receptor-mediated endocytosis and subsequent mRNA delivery and expression. In contrast, 293T cells lacked functional expression of these glycan receptors; consequently, no significant difference in transfection efficiency was observed between Ben and Ben-Man LNPs in this non-DC control cell line.

### 3.3. Efficient Endosomal Escape and Transfection Efficiency of Ben-Man LNPs

To assess endosomal escape, mRNA-encapsulated LNPs were co-incubated with calcein for 2 h. Given the membrane-impermeable nature of calcein, its cytosolic appearance serves as a functional indicator of endosomal membrane disruption [53]. As shown in Figure 3D, because of efficient endosomal escape, Ben-Man LNPs induced pronounced calcein diffusion into the cytosol of DC2.4 cells, while calcein in Man LNPs and BXA LNPs remained aggregated at the endosomal membrane or cell membrane, without showing any diffusion. Furthermore, ζ-potential measurements revealed that Ben-Man LNPs underwent pH-dependent surface charge modulation: their ζ-potential shifted from near-neutral at physiological pH to significantly positive under acidic conditions (Figure 3E). This behavior may arise from the acid-triggered dissociation of the Ben-mPEG_2000_ shielding layer, which exposes protonatable primary amine groups (-NH_2_). Subsequent protonation yields -NH_3_^+^ moieties, accounting for the observed increase in positive surface charge. We proposed that this pH-responsive charge reversal enhanced electrostatic interactions with negatively charged endosomal membranes, thereby synergistically promoting membrane destabilization alongside ionizable lipids.

Owing to its dual advantages—DC-specific targeting ability (like a ligand key to make ligand–receptor “unlocking”) and enhanced endosomal escape capability (resulting in “wall” membrane rupture)—Ben-Man LNPs demonstrated the highest mRNA delivery efficiency and subsequent protein expression (as apple juice) among the tested formulations (Figure 3F). Subsequent analysis demonstrated that Ben-Man LNPs achieved superior transfection efficiency in both DC lines (DC2.4 and BMDCs) and 293T cells (Figure 3G). In DCs, the relative fluorescence intensity of mCherry protein expressed following transfection with Ben-Man LNPs was significantly higher than that observed with either Ben LNPs or Man LNPs, highlighting a synergistic enhancement conferred by combining mannose-mediated DC targeting with pH-assisted endosomal escape. Notably, Man LNPs exhibited approximately 1.5-fold greater fluorescence intensity than BXA LNPs in DCs but showed no statistically significant difference in 293T cells, further validating the DC-selective advantage of mannose functionalization for mRNA delivery. Moreover, Ben LNPs outperformed BXA LNPs in terms of transfection efficiency across all tested cell types, confirming the functional benefit of the pH-enhanced endosomal escape mechanism. The integration of both strategies—pH-mediated endosomal disruption and mannose-directed DC targeting—yielded superior fluorescence intensity for Ben-Man LNPs across all groups. Notably, we previously demonstrated that this class of pH-responsive LNPs, mediated by free primary amine (−NH_2_) as the responsive functional group, could complete mRNA endosomal escape within 4 h, activating the early endosomal escape pathway (Rab5A) to promote membrane fusion and rupture between early endosomes and the LNPs, thereby enabling mRNA release from early endosomes into the cytoplasm for expression [47]. This established mechanism corroborates the enhanced endosomal escape inferred from the increased surface charge and reduced PEG steric hindrance of Ben-Man LNPs in the present work.

### 3.4. STING Pathway Activation and Immunostimulatory Effects of Ben-Man LNPs

To evaluate the effect of STING pathway activation and the immune activation potential of Ben-Man LNPs, we utilized STING-R283S mRNA as a payload, verified the protein expression of STING and IRF3, as well as their phosphorylation, and assessed DC and T-cell activation via flow cytometry. To verify the antitumor immune effect, we quantified cytokine levels via ELISA and measured Panc02 tumor cell viability following immune killing using both CCK-8 and LDH assays.

**Figure 4:**
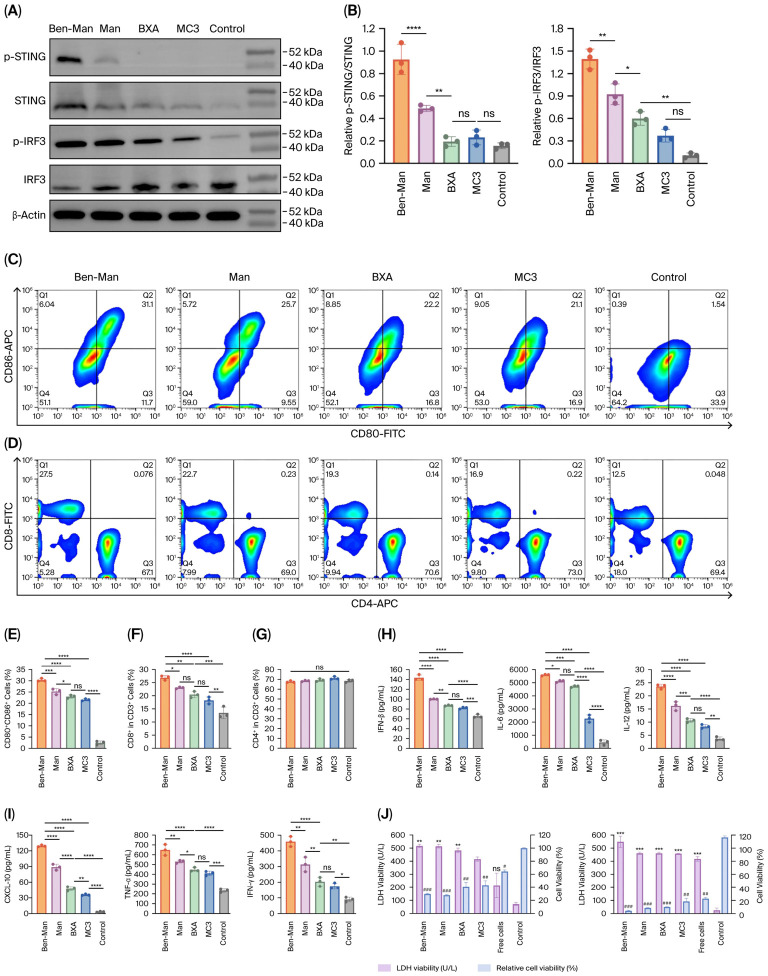
Immune activation in vitro. (**A**) Activation of the STING pathway in DC2.4 cells after treatment with the indicated STING-R283S mRNA-encapsulated LNPs, as shown by Western blot, and (**B**) quantification (n = 3). Flow analysis of (**C**) mature DCs and (**D**) T cells after treatment with the indicated STING-R283S mRNA-encapsulated LNPs for 24 h (n = 3). Flow analysis and quantification of (**E**) mature DCs and (**F**,**G**) T cells after co-incubation with Panc02 cells treated with indicated STING-R283S mRNA-encapsulated LNPs for 24 h (n = 3). (**H**,**I**) Analysis of antitumor cytokines in vitro by enzyme-linked immunosorbent assay (n = 3). (**J**) Evaluation of cytotoxic killing ability and LDH expression level after treatment with indicated STING-R283S mRNA-encapsulated LNPs for 24 and 48 h (n = 3, * and # were compared with the control). A one-way ANOVA with Tukey test was used to assess significant differences (^#^ *p* or * *p* < 0.05; ^##^ *p* or ** *p* < 0.01; ^###^ *p* or *** *p* < 0.001, **** *p* < 0.0001, ns: no significant difference). Data are presented as mean ± SD.

Notably, as shown in Figure 4A,B, compared with the MC3 LNPs (commercial LNPs), both the Ben-Man LNP and Man LNP groups exhibited significantly elevated p-STING expression (*p* < 0.01), with the Ben-Man LNP group showing the highest level—approximately 1.91-fold higher than that in the Man LNP group (*p* < 0.0001). Consistent with STING pathway activation, p-IRF3 protein levels increased across all treatment groups relative to the control, confirming successful functional engagement of the STING-R283S pathway. Importantly, the Ben-Man LNP group exhibited the greatest p-IRF3 induction, suggesting that Ben-Man LNPs potentiate STING-R283S-driven signaling in DCs. This enhanced signaling is expected to amplify the production of IFN-I, thereby promoting DC maturation and priming robust antitumor T-cell responses. Moreover, transfection of STING mRNA and p-STING mRNA mediated by Ben-Man LNP was the strongest across all groups, indicating the effectiveness of the combined strategy of DC targeting and pH responsiveness. There was no significant difference in STING protein expression between the Man LNP group and the BXA LNP group, but p-STING protein expression was significantly increased (*p* < 0.01). We believe that this is due to activation of the STING pathway leading to STING phosphorylation, which may be more robust in the Man LNP group, resulting in greater conversion of STING to p-STING. Consequently, total STING expression showed no significant difference compared to that in the BXA LNP group, while p-STING was markedly upregulated (Figure 4A and Figure S6).

Next, we measured the activation level of immune cells after co-incubation with Panc02 cells and LNPs. The Ben-Man LNP group significantly increased the number of mature DCs, exhibiting an approximately 1.5-fold increase compared with the MC3 LNP group (Figure 4C,E). The maturation rate of DCs in the Ben-Man group was markedly higher than that in the Man LNP group (CD80^+^CD86^+^: 30.33% ± 0.67% vs. 25.27% ± 1.31%), reflecting superior mRNA delivery performance through pH-enhanced mRNA endosomal escape. Moreover, mannose-targeted Man LNPs induced a 1.1-fold increase in the proportion of mature DCs compared with BXA LNPs. We next evaluated the impact of different LNP formulations on T lymphocyte activation, with particular focus on CD4^+^ T helper and CD8^+^ cytotoxic T effector populations. As shown in Figure 4D–G, while no statistically significant variation in CD4^+^ T-cell frequency was observed across treatment groups, Ben-Man LNPs loaded with STING-R283S mRNA induced a robust expansion of CD8^+^ T effector cells—increasing their abundance by approximately 1.5-fold relative to MC3-based LNPs and 2.0-fold compared to untreated controls. These findings suggested that Ben-Man LNPs effectively promote the differentiation and/or proliferation of antigen-specific CD8^+^ T cells, thereby skewing the adaptive immune response toward a potent, effector-dominated antitumor phenotype.

To assess the antitumor immune activity of the LNPs, we quantified cytokine levels (IFN-β, IL-6, IL-12, CXCL10, IFN-γ, TNF-α) in culture supernatants following exposure to STING-R283S mRNA-loaded LNPs. Ben-Man LNPs induced significantly stronger antitumor immune responses than BXA LNPs, as evidenced by the increased levels of IFN-β (1.6-fold), IL-6 (1.1-fold), IL-12 (2.2-fold), CXCL10 (2.7-fold), TNF-α (1.4-fold), and IFN-γ (2.2-fold) (Figure 4H,I), suggesting the synergistic effect of DC targeting and pH-enhanced endosomal escape. Importantly, cytokine induction by Ben-Man LNPs exceeded that observed with Man LNPs, providing direct functional evidence supporting the enhanced endosomal escape conferred by our pH-responsive benzamide bond linkage.

Next, we assessed the immunologically mediated antitumor activity of Ben-Man LNPs using complementary functional assays: the CCK-8 assay to quantify tumor cell viability and the LDH release assay to measure cytotoxic immune effector activity, both serving as indirect indicators of immune-driven tumor cell killing. As shown in Figure 4J, Ben-Man LNPs significantly activated antitumor immunity compared with the control group, resulting in the lowest cell viability and the highest LDH levels among all treatment groups within 24 and 48 h. As explained earlier (Introduction), the release of IFN-I can induce the secretion of DAMPs from a subset of Panc02 cells; these DAMPs are then captured and presented by DCs as antigens, thereby activating T cells to mediate antitumor immune killing [18,31,32].

### 3.5. Ben-Man LNPs/STING-R283S Enhanced Intratumoral DC Targeting and Induced Immune Responses with αPD-L1 to Inhibit Pancreatic Cancer Growth

The uptake and targeting of Cy5-labeled mRNA-loaded LNPs by tumor-infiltrating DCs were assessed via cryo-sectioning followed by immunofluorescence staining (Figure S7). Twelve hours after intratumoral injection, the co-localization coefficients between CD11c^+^ DCs and Cy5-LNPs were significantly higher in the mannose-modified LNP groups: 0.81 ± 0.09 for Ben-Man and 0.80 ± 0.17 for Man, compared with 0.55 ± 0.09 for BXA and 0.46 ± 0.08 for MC3 (*p* < 0.01). Mannose modification markedly enhances LNP delivery specificity and internalization by intratumoral DCs, thereby establishing a critical prerequisite for efficient STING pathway activation and subsequent DC functional maturation.

**Figure 5:**
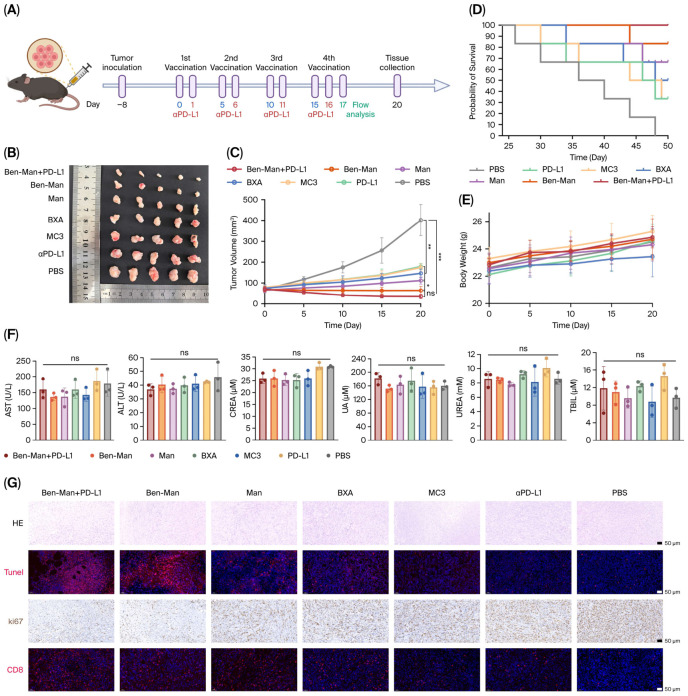
Antitumor efficacy and safety profile of therapeutic interventions in the Panc02 syngeneic pancreatic cancer model. (**A**) Experimental timeline illustrating the treatment schedule in Panc02 tumor-bearing C57BL/6 mice. (Created in BioRender. Weiwei, J. (2026) https://BioRender.com/9a2ycqb (accessed on 5 June 2026)) (**B**,**C**) Representative photographs and quantitative tumor volume measurements of subcutaneous Panc02 tumors across treatment groups (n = 5 per group). (**D**) Survival analysis showing prolonged survival in mice receiving combination therapy (n = 6 per group). (**E**) Longitudinal monitoring of body weight change as an indicator of systemic tolerability (n = 5). (**F**) Comprehensive serum biochemistry panel including ALT, AST, CREA, TBIL, UREA, and UA to evaluate hepatic and renal function (n = 3). (**G**) Multiplex histopathological evaluation of excised tumor tissues: H&E for overall architecture and necrosis, TUNEL for apoptotic nuclei, Ki67 for proliferative index, and CD8 immunostaining for cytotoxic T-cell infiltration; scale bar = 50 µm. Statistical significance was determined using one-way ANOVA with Tukey’s multiple comparisons test (* *p* < 0.05, ** *p* < 0.01, *** *p* < 0.001, ns: no significant difference); all data are expressed as mean ± SD.

The antitumor activity of Ben-Man LNPs delivering STING-R283S mRNA was evaluated in a pancreatic tumor model. Panc02 cells (1 × 10^6^) were implanted subcutaneously on day 0, and treatment commenced once tumors reached ~80–100 mm^3^ (designated as day 8). As outlined in Figure 5A, mice received intratumoral injections of Ben-Man/STING-R283S mRNA on days 8, 13, 18, and 23, while concurrent αPD-L1 antibody (100 μg, i.p.) was administered on days 1, 6, 11, and 16 to counteract T-cell exhaustion. As shown in Figure 5B,C, the combination of Ben-Man LNPs and αPD-L1 induced profound tumor regression—significantly outperforming monotherapies and control groups. Tumor growth kinetics revealed aggressive progression in the PBS-treated cohort, whereas Ben-Man alone markedly suppressed tumor expansion; this effect was further potentiated by αPD-L1 co-administration. Notably, the Ben-Man + αPD-L1 regimen conferred complete survival protection within 50 days (Figure 5D). Moreover, no significant body weight loss was observed in any treatment group (Figure 5E), indicating minimal systemic toxicity and a favorable safety profile.

Histopathological evaluations (H&E staining) of major organs (heart, liver, spleen, lung, and kidney) revealed no treatment-related abnormalities compared with the PBS group (Figure S8). Furthermore, serum biochemical parameters (e.g., ALT, AST, and UA levels) remained within normal physiological ranges across all treatment groups (Figure 5F). Collectively, these findings demonstrate that the therapeutic regimen exhibited a favorable safety profile, with no evidence of systemic toxicity or organ damage. Histopathological assessment of tumor tissues through H&E staining, Ki67 immunohistochemistry, and the TUNEL assay revealed extensive nuclear condensation, chromatin fragmentation, and widespread apoptotic cell death in both Ben-Man and Ben-Man + αPD-L1 groups, with significantly enhanced cytotoxic effects in the combination cohort (Figure 5G). Moreover, multiplex immunofluorescence analysis showed markedly increased infiltration, proliferation, and effector differentiation of CD8^+^ T cells within the tumor microenvironment following Ben-Man + αPD-L1 therapy, reflecting a robust and functionally active antitumor immune response (Figure 5G). These findings provide compelling evidence for the potent antitumor immune responses elicited by Ben-Man LNPs encapsulated with STING-R283S mRNA, particularly when combined with αPD-L1 therapy through an integrated strategy of DC-targeting and pH-mediated promoted endosomal escape.

### 3.6. Ben-Man LNP/STING-R283S mRNA Promotes Immune Activation in Pancreatic Cancer

**Figure 6:**
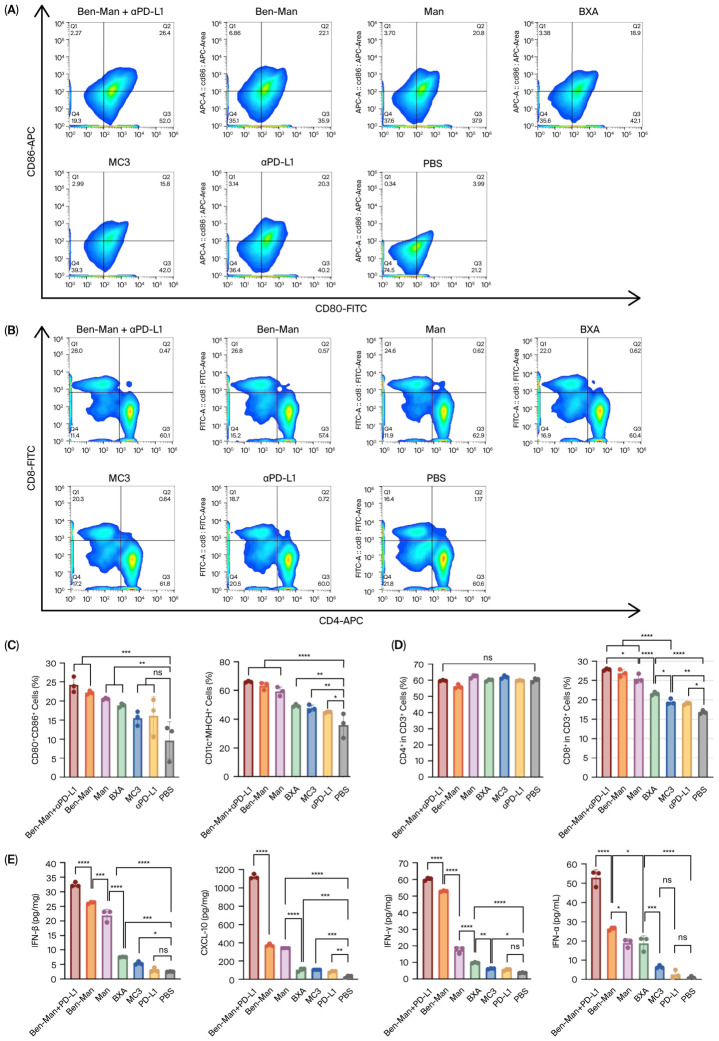
In vivo immune activation profile induced by therapeutic interventions in the Panc02 pancreatic tumor model. (**A**,**B**) Flow cytometric assessment of activated DCs and T cells within tumor tissues. (**C**,**D**) Flow cytometric quantitative analysis of activated DC and T cells (n = 3). (**E**) Multiplex ELISA quantification of immunostimulatory mediators (n = 3). Statistical significance was determined using one-way ANOVA with Tukey’s post hoc test (* *p* < 0.05, ** *p* < 0.01, *** *p* < 0.001, **** *p* < 0.0001, ns: no significant difference); all results are presented as mean ± SD.

Flow cytometry of tumor-infiltrating immune cells on day 17 revealed that the Ben-Man + αPD-L1 group induced the most pronounced DC activation—evidenced by elevated expression of CD80, CD86, and MHC-II—among all treatment cohorts (Figure 6A,C). Although vaccination with all LNP formulations increased intratumoral CD8^+^ T-cell frequencies relative to PBS-treated controls, the combination regimen drove significantly higher accumulation and functional priming of antigen-specific CD8^+^ T lymphocytes (Figure 6B,D).

By day 20, tumor homogenates were analyzed via ELISA for key immunomodulatory cytokines and chemokines, including IFN-β, IFN-γ, TNF-α, and CXCL10. All vaccine groups exhibited upregulated cytokine production compared with controls; however, the Ben-Man + αPD-L1 group displayed markedly superior induction across all measured mediators (Figure 6E), corroborating a potent, synergistic enhancement of innate and adaptive antitumor immunity.

**Figure 7:**
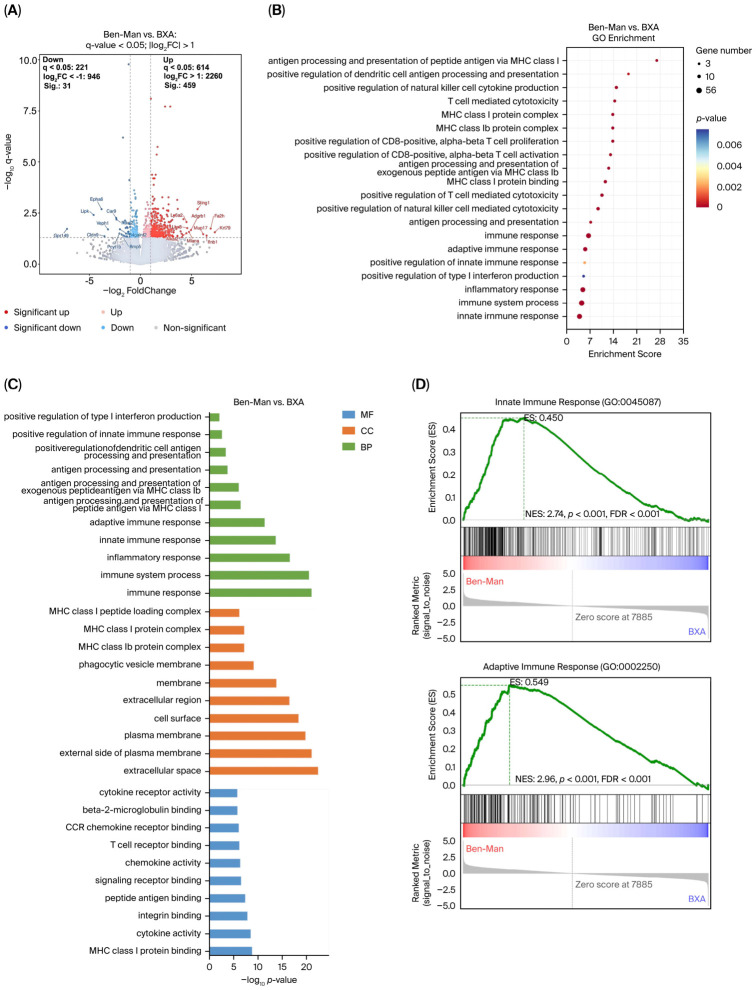
RNA sequencing analysis of tumor tissue. (**A**) Volcano plot showing the differences in gene expression between the Ben-Man and Control (BXA LNP) groups in RNA sequencing (n = 3). (**B**,**C**) Gene Ontology (GO) analysis and (**D**) GSEA of the differentially expressed genes between the Ben-Man and control groups (n = 3, red: up-regulation, blue: down-regulation).

**Figure 8:**
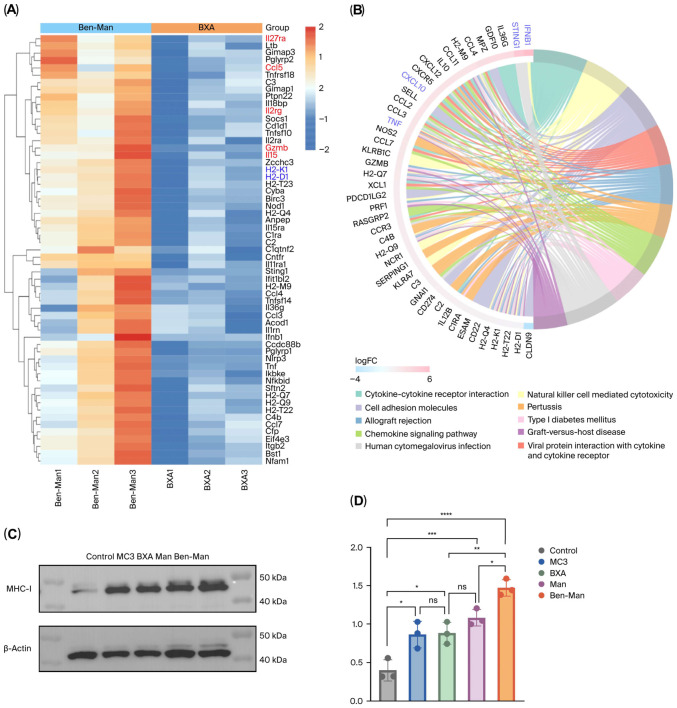
RNA sequencing analysis of tumor tissue. (**A**) Heatmap analysis and (**B**) KEGG analysis of the differentially expressed genes between the Ben-Man and control groups (n = 3). (**C**) Western blot analysis of MHC-I expression in vitro in BMDCs and (**D**) quantitative analysis (n = 3). A one-way ANOVA with Tukey test was used to assess significant differences (* *p* < 0.05; ** *p* < 0.01; *** *p* < 0.001; **** *p* < 0.0001, ns: no significant difference). Data are presented as mean ± SD.

We performed RNA sequencing on Panc02 pancreatic tumor tissues treated with either Ben-Man LNPs or BXA LNPs (control), using the Illumina platform, to further validate the immunomodulatory role of the Ben-Man LNP, which is a dual-functional nanocarrier engineered for DC targeting and pH-enhanced endosomal escape. The Ben-Man LNP group exhibited differential expression of 459 upregulated genes and 31 downregulated genes compared with the control group (Figure 7A). Gene Ontology (GO) enrichment analysis revealed that these differentially expressed genes were significantly enriched in biological processes critical to antitumor immunity, including innate immune response, adaptive immune response, inflammatory response, type I IFN production, and antigen processing and presentation (Figure 7B,C). Consistent with this, Gene Set Enrichment Analysis (GSEA) confirmed robust in vivo activation of both innate and adaptive immune pathways in the Ben-Man LNP group with NES > 2 and FDR < 0.01 (Figure 7D). Gene heatmap visualization further demonstrated the marked upregulation of key immune-regulatory and pro-inflammatory genes, including GzmB, IL27ra, IL2rg, IL15, and CCL5, in the Ben-Man LNP group (Figure 8A). Kyoto Encyclopedia of Genes and Genomes (KEGG) pathway analysis corroborated these findings, showing significant enrichment in IFN-I signaling and immune regulation pathways, with upregulated expression of core mediators such as IFN-β1, STING1, CXCL10, and TNF (Figure 8B). Collectively, these transcriptomic data substantiated that Ben-Man LNPs, when delivering STING-R283S mRNA, effectively activated the innate immune axis and reprogrammed the immunosuppressive pancreatic TME. Notably, the gene heatmap analysis also revealed pronounced upregulation of H2-K1 and H2-D1, which are classical MHC class I genes essential for endogenous antigen presentation to CD8^+^ T cells (Figure 8A). Ben-Man LNP/STING-R283S enhanced the antigen-presenting capacity of DCs within the TME. To experimentally confirm this inference, we assessed MHC-I protein expression in BMDCs and observed significant upregulation following Ben-Man LNP/STING-R283S treatment (Figure 8C,D), thereby functionally validating enhanced antigen presentation after Ben-Man LNP/STING-R283S treatment.

## 4. Discussion

This study developed a novel pH-responsive PEG lipid based on benzamide bonds and engineered DC-targeted/pH-responsive Ben-Man LNPs to overcome the endosomal escape bottleneck in mRNA delivery via LNPs and to address the severely suppressed STING signaling pathway in pancreatic cancer. Under physiological endosomal conditions (pH5.5), the pH-responsive PEGylated lipid undergoes cleavage, releasing PEG_2000_ while retaining PEG_400_ grafted onto the LNP surface with exposed amine functional groups. On one hand, the exposed amines become further protonated in acidic environments, increasing the zeta potential of the LNPs and thereby facilitating membrane fusion mediated by ionizable lipids. On the other hand, cleavage of the pH-responsive PEGylated lipid reduces steric hindrance between the LNPs and the endosomal membrane, promoting membrane fusion and enhancing mRNA escape from endosomes.

We selected the STING-R283S mutant (whose human homolog is R284S) over wild-type STING due to its unique signaling properties, which overcome two major obstacles in pancreatic cancer immunotherapy: direct toxicity of conventional agonists to T cells and tumor-mediated suppression of the STING pathway [35]. The R283S mutation resides in the C-terminal tail of the STING protein, a region critical for TBK1 recruitment and IRF3 phosphorylation [54,55]. This acquired gain-of-function mutation causes STING to spontaneously translocate from the endoplasmic reticulum to the Golgi apparatus even in the absence of ligand stimulation, thereby continuously activating the TBK1-IRF3 pathway and sustaining IFN-I production without cyclic dinucleotides (CDNs) [54,55]. Crucially, conventional CDN-based STING agonists not only enter antigen-presenting cells but also penetrate T lymphocytes, where they trigger endogenous STING activation, leading to apoptosis via IRF3- and NF-κB-dependent pathways and causing severe T-cell exhaustion [56]. In our strategy, STING-R283S is delivered as mRNA encapsulated within DC-targeted LNPs, restricting STING expression primarily to DCs. This spatially confined activation avoids direct STING signaling in T cells, thereby reactivating antitumor immunity without inducing lymphocyte proliferation inhibition.

More importantly, pancreatic tumors often develop resistance to STING agonists by upregulating ecto-nucleotide pyrophosphatase/phosphodiesterase 1 (ENPP1), which hydrolyzes the endogenous ligand 2′,3′-cGAMP, or by epigenetically silencing the cGAS gene [57]. Since STING-R283S transmits signals independently of ligands, it entirely bypasses the need for cGAS-generated or exogenously added cyclic dinucleotides [35], rendering this pathway insensitive to ENPP1-mediated degradation and cGAS silencing. Collectively, these characteristics make STING-R283S a unique, highly effective, and tumor-resistant STING module ideally suited for immunotherapy against pancreatic cancer.

Moreover, the Ben-Man LNP developed in this study exhibits significant DC-targeting efficacy. However, due to differences among DC subsets, the heterogeneity in mannose receptor expression across various DC populations may affect the targeting efficiency of our mannose-functionalized LNPs. Mannose receptors are unevenly distributed among different DC subsets, being predominantly expressed on conventional type 2 dendritic cells (cDC2) and monocyte-derived dendritic cells (moDCs), while their expression is low or even negligible on conventional type 1 dendritic cells (cDC1) and plasmacytoid dendritic cells (pDCs) [58]. Therefore, based on the relevant literature [58], our mannose-targeting strategy is expected to favor transfection of cDC2 and moDCs, with minimal impact on cDC1. Nevertheless, this inherent subset preference does not necessarily limit the antitumor efficacy of this approach. First, both cDC2 and moDCs are fully capable of initiating robust antitumor CD4^+^ T-cell responses, which in turn provide critical support for the activation and expansion of tumor-specific CD8^+^ cytotoxic T lymphocytes [59]. Second, the IFN-I environment generated by STING-R283S in mannose receptor-expressing DCs can activate neighboring cDC1 cells via paracrine signaling, thereby enhancing their capacity for cross-presentation of tumor antigens [60]. Consistent with these findings, our in vivo data demonstrate strong tumor-specific CD8^+^ T-cell responses and significant tumor regression, indicating that despite the biased targeting, an effective antitumor immune cascade has been successfully initiated. Thus, our mannose-LNP platform, by targeting a biologically relevant subset of DCs, is sufficient to orchestrate effective antitumor immunity.

Notably, the heterogeneous expression of mannose receptors across different DC subsets may not only dictate differential nanoparticle uptake but also skew subsequent antigen processing and functional maturation. For example, the preferential targeting of CD103^+^ migratory DCs, which exhibit high cross-presentation capacity, would be expected to enhance MHC-I-restricted antigen presentation and the subsequent priming of tumor-specific CD8^+^ T cells. Conversely, nanoparticle internalization by less immunogenic DC subsets might lead to tolerogenic maturation profiles or suboptimal T-cell activation. Our observations of robust STING R283S-mediated immunization and tumor growth inhibition indirectly suggest a favorable targeting pattern, but future studies incorporating single-cell analysis of DC maturation markers (CD80, CD86, CD40), direct visualization of antigen cross-presentation (e.g., SIINFEKL-H-2Kb complexes), and ex vivo T-cell recall assays are warranted to fully dissect these mechanisms.

However, this study still has several limitations. First, the effects of STING-R283S and wild-type STING, as well as the underlying mechanisms associated with the mutation—such as whether the amplification of STING signaling by STING-R283S is mutation-dependent and how the mutation affects downstream signaling pathways (e.g., IRF3, TBK1, and IFN-I)—have not been investigated. Therefore, in future studies, we plan to explore the mechanism by which mutated STING-R283S enhances antitumor immune responses, aiming to more thoroughly elucidate the differences between STING-R283S and wild-type STING at the mechanistic level, including their signaling advantages and resistance to tumor-mediated suppression. This will reveal the critical role of STING-R283S in activating the STING pathway within the cold tumor microenvironment, providing new insights and perspectives for future STING-based cancer immunotherapies. Second, this study did not address the impact of “heterogeneity in mannose receptor expression across different DC subtypes on targeting efficiency,” which represents a crucial supporting dataset for mannose-targeted DC strategies. Recognizing this subtype-specific preference will help us interpret our findings more precisely and guide the design of next-generation nanovaccines targeting DCs. We intend to focus future research on summarizing how heterogeneity among different DC subsets influences mannose-targeting efficacy. Finally, this study only validated the therapeutic effect of Ben-Man LNP-delivered mRNA combined with αPD-L1 in a syngeneic mouse subcutaneous tumor model. To further investigate the strength of immune responses induced by this LNP system and to better understand the advantages of combination therapy over monotherapy, future studies should evaluate the treatment’s efficacy in orthotopic and metastatic tumor models.

## 5. Conclusions

In summary, we successfully developed a mannose-modified and pH-responsive mRNA-LNP that achieves selective delivery to DCs and enables rapid, efficient endosomal escape, thereby markedly enhancing STING-R283S-mediated activation of the STING pathway and potentiating DC antigen presentation. Ben-Man LNPs encapsulating STING-R283S mRNA were rapidly internalized by DCs and released into the cytosol, supporting robust mRNA translation within 4 h. Using quantitative assays for endosomal membrane permeabilization and pH-dependent zeta potential measurements, we confirmed that the cleavable PEGylated lipid (Ben-mPEG_2000_) critically facilitates endosomal rupture and subsequent DC activation. In vivo therapeutic evaluation revealed that Ben-Man LNPs/STING-R283S mRNA significantly outperformed the clinically benchmarked MC3-based LNP formulation (used in FDA-approved mRNA vaccines), especially when combined with αPD-L1. Based on the RNA sequencing results, Ben-Man LNPs/STING-R283S mRNA successfully reversed the immunosuppressive microenvironment of pancreatic cancer, transforming the tumor from “cold” to “hot”.

## Data Availability

Data presented in this study is contained within the article and Supplementary Material. Further inquiries can be directed to the corresponding authors.

## Supplementary Materials

The following supporting information can be downloaded at https://www.mdpi.com/article/10.3390/pharmaceutics18060760/s1: Figure S1: Molecular structure identification of BXA2-8 ionizable lipid by HRMS; Figure S2: Molecular structure identification of BXA2-8 by ^1^H NMR; Figure S3: The synthetic route of Ben-mPEG_2000_; Figure S4: Molecular structure identification of Ben-mPEG_2000_ by ^1^H NMR; Figure S5: Grafting efficiency of Ben-mPEG_2000_ with different molar ratios; Figure S6: Quantitative analysis of STING and p-STING protein expression by Western blot. Figure S7: The co-localization of Cy5-LNPs with DCs in lymph nodes; Figure S8: H&E staining analysis of important tissues; Table S1: The physicochemical properties of LNPs.

## Abbreviations

The following abbreviations are used in this manuscript:

LNPs	Lipid nanoparticles
mRNA	Messenger RNA
STING	Stimulator of interferon genes
ICIs	Immune checkpoint inhibitors
TME	Tumor microenvironment
DCs	Dendritic cells
IFN-I	Type I interferon
DAMPs	Damage-associated molecular patterns
BXA	N1, N3, N5-tris(2-aminoethyl)benzene-1,3,5-tricarboxamide
RES	Reticuloendothelial system
Chol	Cholesterol
DSPC	1,2-Dioctadecanoyl-sn-glycero-3-phosphocholine
DOPE	1,2-Dioleoyl-sn-glycero-3-phosphoethanolamine
HPR	Anti-rabbit IgG H&L
TNBS	Tetrazolium bromide
BMDCs	Bone marrow-derived dendritic cells
SLCs	Splenic lymphocytes
CLSM	Confocal laser scanning microscopy
CCK-8	Cell Counting Kit-8
ELISA	Enzyme-linked immunosorbent assay
CTL	Cytotoxic T lymphocyte
LDH	Lactate dehydrogenase
H&E	Hematoxylin and eosin
ECL	Enhanced chemiluminescence
^1^H NMR	Nuclear magnetic resonance (1H NMR) spectroscopy
HRMS	High-resolution mass spectrometry
CDN	Cyclic dinucleotides
ENPP1	Ecto-nucleotide pyrophosphatase/phosphodiesterase 1
cDC1	Type 1 dendritic cells
cDC2	Type 2 dendritic cells
moDCs	Monocyte-derived dendritic cells
pDCs	Plasmacytoid dendritic cells
EGFR	Epidermal growth factor receptor
