# Supplementary material for: Engineered Lipid Nanoparticles with Promoted Endosomal Escape and R283S-Mediated Stimulator of Interferon Genes (STING) Activation for Pancreatic Cancer Immunotherapy
Source: Pharmaceutics. 2026 Jun 21;18(6):760. doi: 10.3390/pharmaceutics18060760 (PMC13307127; doi:10.3390/pharmaceutics18060760)
Supplement: Supplementary file 1 [file pharmaceutics-18-00760-s001.zip › pharmaceutics-4368348-supplementary.pdf]

*Supplementary Materials*

# Engineered Lipid Nanoparticles with Promoted Endosomal Escape and R283S-Mediated Stimulator of Interferon Genes (STING) Activation for Pancreatic Cancer Immunotherapy

**Sizhen Wang [1,†], Qiwei Tai [1,†], Kehui Wang [1], Jianyu Zheng [1], Beibei Guo [1], Feng Yang [1,*] and Chen Wang [2,*]**

[1] School of Pharmacy, Naval Medical University, Shanghai 200433, China; wsz08242546@163.com (S.W.); m13764705533_1@163.com (Q.T.); piu630qwq@163.com (K.W.); zhengjianyu2024@163.com (J.Z.); bbguo1994@163.com (B.G.)
[2] Department of Oncology, Xinhua Hospital, School of Medicine, Shanghai Jiao Tong University, Shanghai 200092, China
* Correspondence: yangfeng1008@126.com (F.Y.); 7220030151@shsmu.edu.cn (C.W.)
† These authors contributed equally to this work.

## Supplementary Text

*1.1. Synthesis of benzene-1,3,5-tricarboxamide ionizable lipids (BXA2-8)*

The BXA ionizable lipid (BXA2-8) was prepared according to a previous study [1]. In brief, 1.88 mmol of 1,3,5-benzotrioyl chloride was weighed and dissolved in 30 mL of dichloromethane. Subsequently, 10 mL of pyridine was added under ice-bath conditions, and the mixture was stirred thoroughly. Then, 7.52 mmol of BoC-1,2-ethylenediamine was dissolved in 30 mL of dichloromethane and added drop by drop to the above solution system while it was being agitated. The resulting mixture was stirred at room temperature for 24 h. Then, it was washed three times with saturated sodium bicarbonate solution and saturated sodium chloride solution; it was then extracted with dichloromethane, and the organic phase was collected. The organic phase was dried with anhydrous magnesium sulfate for 1 h and filtered, and the organic solvent was removed by rotary evaporation to obtain the crude product. The crude product was then purified by column chromatography (dichloromethane: methanol = 20:1) to obtain the final product, BOC-1,3,5-phenyltriformamide-2.

A total of 1.48 mmol of BoC-1,3,5-phenyltriformamide-2 was dissolved in 100 mL of dichloromethane. Under ice-bath conditions, 26.64 mmol of trifluoroacetic acid (TFA) was added, and the mixture was stirred at room temperature for 15 - 24 hours. After deprotection was completed, the organic solvent was removed from the system by rotary evaporation. Then, methanol was added to redissolve the residue, and the methanol was concentrated again by rotary evaporation. Ten times the volume of ethyl acetate was added for recrystallization. The crystal precipitate was collected by centrifugation, and the residual ethyl acetate was removed by rotary evaporation to obtain 1,3,5-phenyltriformamide 2.

A total of 1 mmol of 1,3,5-phenyltriformamide-2 was weighed and dissolved in 100 mL of tetrahydrofuran. Under nitrogen protection, 5 mmol of triethylamine solution was added. After stirring at room temperature for 1 h, 9 mmol of n-octanal and 5 mmol of sodium triacetoxyborohydride were added, and the mixture was stirred at room temperature for 48 h under nitrogen protection. After the reaction, the mixture was washed 3 times with saturated sodium bicarbonate solution and saturated sodium chloride solution; it was then extracted with dichloromethane, and the organic phase was collected, dried with anhydrous magnesium sulfate for 1 h, and filtered. The organic solvent was rotary-evaporated to obtain the crude product. The final product BXA2-8 was obtained by over-

column purification (dichloromethane: methanol = 20:1). The molecular structure and mass-to-charge ratio of the final products were confirmed by HRMS **(Figure S1)** and $^{1}$H-NMR **(Figure S2)**. The mass-to-charge ratio (m/z) obtained by HRMS was 1009.9512, matching the theoretical value of 1009.95 **(Figure S1)**. These results confirm the successful synthesis of compound BXA2-8.

### *1.2. Synthesis of pH-responsive benzamide-based mPEG$_{2000}$ lipid (Ben-mPEG$_{2000}$)*

The synthetic route is described in **Figure S3**. Briefly, 0.185 mmol of BOCNH-PEG$_{400}$-COOH was dissolved in 3 mL of dichloromethane, and then 0.37 mmol of EDCI, 0.463 mmol of DIPEA and 0.018 mmol of DMAP were added. The mixture was activated in an ice bath for 1 hour. Afterwards, 0.204 mmol of 1,2-dimyristoylglycerol (DMG, purchased from Xi'an Ruixi Biological Technology Co., Ltd) was added and stirred at room temperature for 48 hours. The mixture was washed three times with 0.002 M citric acid solution, saturated sodium bicarbonate solution and saturated sodium chloride solution, in that order, and then extracted with dichloromethane. The organic phase was collected and purified by column chromatography (dichloromethane: methanol = 50 : 1). **Compound 1** (BOCNH-PEG$_{400}$-DMG) was obtained.

A total of 1 mmol of Compound 1 was dissolved in an appropriate amount of dichloromethane, and 10 mmol of TFA was added under an ice bath. The reaction was carried out at room temperature to remove BOC protection. The reaction progress was monitored by ultraviolet spectrophotometry. After complete deprotection, saturated sodium bicarbonate solution was gradually added dropwise to the reaction mixture in an ice bath for neutralization; then the solution was extracted with dichloromethane, and the organic phase was collected and evaporated under reduced pressure to obtain **Compound 2** (DMG-PEG$_{400}$-$NH_2$).

A total of 1 mmol Compound 2 was dissolved in 2 ml of N,N-dimethylformamide (DMF), and 1.5 mmol of benzaldehyde-PEG$_{2000}$ (purchased from Xi'an Ruixi Biological Technology Co., Ltd) was added. The mixture was reacted at room temperature for 24 hours. The solution was then subjected to dialysis in deionized water and freeze-drying to obtain the target compound, Ben-mPEG$_{2000}$. The molecular structure of Ben-mPEG$_{2000}$ was confirmed by $^{1}$H NMR **(Figure S4)**, indicating that the synthesis of the compound Ben-mPEG$_{2000}$ was successful.

### *1.3. Measurement of Ben-mPEG$_{2000}$ Modification Efficiency*

Ben-mPEG$_{2000}$ modification efficiency was quantified by detecting the remaining primary amine groups using TNBS [1]. The Ben-Man LNP solution was diluted with 0.1 M borate buffer (pH 9.5) and treated with 1% TNBS for 60 min. Following treatment, 10% Triton X-100 was added to a final concentration of 1.0% (w/v), and absorbance was measured at 420 nm, normalized against Compound 2. Modification efficiency was calculated as follows:

Modification Efficiency (ME%) = $1 - (A_U - A_{U0}) / (A_T - A_{T0}) \times 100\%$

$A_U$ is the absorbance of the Ben-Man LNP solution, $A_{U0}$ is the blank solvent absorbance, $A_T$ is the absorbance of Compound 2, and $A_{T0}$ is the corresponding blank solvent absorbance.

### *1.4. Isolation of BMDCs and SLCs*

Isolation of bone marrow-derived dendritic cells (BMDCs) from mice: Six-week-old C57BL/6 mice were euthanized by cervical dislocation and then immersed in 75% ethanol for 5 minutes. The femurs and tibias from the hind limbs were removed under a laminar

flow hood and washed twice with PBS. Using scissors, both ends of the bones were cut near the joints, and bone marrow was flushed out using an injection syringe filled with RPMI-1640 medium. This flushing process was repeated 2–3 times until the marrow cavities turned completely white. The bone marrow suspension was repeatedly pipetted to dissociate all marrow clumps. The cell suspension was filtered through a 70 μm mesh sieve and centrifuged at 1000 rpm for 5 minutes, and the supernatant was discarded. One milliliter of red blood cell lysis buffer was added, gently mixed, and incubated at room temperature for 5 minutes. After lysis, 6 mL of RPMI-1640 medium was added to neutralize the lysis solution, followed by another centrifugation at 1000 rpm for 5 minutes. The supernatant was discarded again. The cell pellet was resuspended in 3 mL of complete RPMI-1640 medium (90% RPMI-1640 + 10% FBS + 1% streptomycin–penicillin mixture + 10 ng/mL IL-4 + 20 ng/mL GM-CSF), the suspension was gently mixed, and the cells were counted. The cell concentration was adjusted to $5\times10^6$ cells/mL with complete RPMI-1640 medium and seeded into 6-well plates for culture.

Six-week-old male C57BL/6 mice were subcutaneously inoculated with $1\times10^6$ Panc02 melanoma cells in the right hind limb and allowed to survive for an additional week before harvesting. The mice were euthanized, immersed in 75% ethanol for 5 minutes, and then dissected under a laminar flow hood. Spleens were removed and placed into a 6-well plate containing 2 mL of complete 1640 medium (90% RPMI-1640 + 10% FBS + 1% streptomycin–penicillin). The spleens were thoroughly ground using a sterile syringe plunger, and the resulting cell suspension was filtered through a 40 μm cell strainer. The tissue debris was centrifuged at 1000 rpm for 5 minutes, and the supernatant was discarded. Cells were resuspended in red blood cell lysis buffer and incubated at room temperature for 5 minutes. Lysis was stopped by adding 3 mL of RPMI-1640 medium, followed by centrifugation at 1000 rpm for 5 minutes. The supernatant was discarded, and cells were resuspended in 5 mL of 1640 medium. A new 15 mL centrifuge tube was prepared with 5 mL of lymphocyte separation solution. The 5 mL cell suspension was gently layered onto the separation solution along the tube wall and centrifuged at 600 × g for 25 minutes at 20°C. After centrifugation, the second layer from the top, a ring-like milky white band, contained purified lymphocytes. This layer was carefully collected into a fresh 15 mL tube, resuspended in 10 mL of RPMI-1640 medium, mixed thoroughly, and centrifuged again at 1000 rpm for 5 minutes. The supernatant was discarded. This washing step was repeated once more. Finally, the purified lymphocytes were resuspended in complete 1640 medium for subsequent experiments.

### *1.5. In vitro flow cytometry analysis*

Flow cytometric analysis of DCs: FSV510 viability dye was diluted 1:1000; CD11c-PE (Clone: N418), CD80-FITC (Clone: 16-10A1), CD86-APC (Clone: GL-1), and MHC-II (Clone: M5/114.15.2) were diluted 1:100. After cell incubation, the 96-well U-bottom plate was centrifuged at 1000 rpm for 5 minutes. Then, 100 μL of diluted FSV510 solution was added, and the cells were incubated at room temperature in the dark for 15 minutes. After incubation, 1 mL of PBS was added to neutralize the dye, followed by centrifugation at 1000 rpm for 5 minutes. Next, 100 μL of the mixed antibody dilution solution was added, and the cells were incubated at room temperature in the dark for 30 minutes. After incubation, 1 mL of PBS was added to neutralize the antibody, followed by another centrifugation at 1000 rpm for 5 minutes. The cells were washed twice with PBS and finally resuspended in 200 μL of PBS for flow cytometry analysis. Single-stained controls and blank controls were included simultaneously.

Flow cytometry analysis of T cells: FSV510 live/dead stain was diluted 1:1000; CD3-PE (Clone: 145-2C11), CD4-APC (Clone: RM4-5), and CD8a-APC780 (Clone: 53-6.7) were diluted 1:100. After cell incubation, the 96-well U-bottom plate was centrifuged at 1000 rpm for 5 minutes. Then, 100 μL of diluted FSV510 solution was added and incubated at room temperature in the dark for 15 minutes. After incubation, the dye was neutralized with 1 mL of PBS, and the solution was centrifuged at 1000 rpm for 5 minutes. Then 100 μL of mixed antibody dilution solution was added and incubated at room temperature in the dark for 30 minutes. After incubation, the antibodies were neutralized with 1 mL of PBS, and the solution was centrifuged at 1000 rpm for 5 minutes. Cells were washed twice with PBS and finally resuspended in 200 μL of PBS for flow cytometry analysis. Single-stained controls and blank controls were included simultaneously.

### *1.6. Immune cell extraction and flow cytometry analysis from tumor tissue*

Digestion solution preparation: A volume of 3.44 mL of RPMI-1640 medium was taken, and 0.4 mL of collagenase IV (10 mg/mL) and 0.16 mL of DNase I (5 mg/mL) were added.

On day 17 after drug administration, tumor tissues from each group of mice were excised, minced, and incubated with 4 mL of digestion solution at 37°C and 100 rpm for 40 minutes. After digestion, the mixture was filtered through a 100 μm cell strainer to remove tissue clumps. The cells were then centrifuged at 1000 rpm for 5 minutes, the supernatant discarded, and the pellet resuspended in 1 mL of PBS. The cells were counted, and the cell concentration was adjusted to $1\times10^6$/mL for later use.

For each group, 100 μL of cell suspension was transferred into a 1.5 mL centrifuge tube, and 0.1 μL of FVS510 (1:1000) was added, followed by gentle pipetting to mix thoroughly. The mixture was incubated at room temperature away from light for 15 minutes. After incubation, 1 mL of PBS was added to neutralize the dye, and the sample was centrifuged at 1000 rpm for 5 minutes. The supernatant was discarded. Then, 100 μL of flow cytometry antibody dilution buffer (CD11c-PE, CD80-FITC, CD86-APC; CD45-APC780, CD3-PE, CD4-APC, CD8a-FITC; all diluted 1:100) was added and incubated at room temperature in the dark for 30 minutes. After incubation, 1 mL of PBS was added to neutralize the antibodies, and the sample was centrifuged again at 1000 rpm for 5 minutes. The supernatant was removed, and the cells were washed once with PBS. Finally, the cells were resuspended in 200 μL of PBS and analyzed by flow cytometry. For each antibody, a single-color control tube was prepared, and negative controls were included simultaneously.

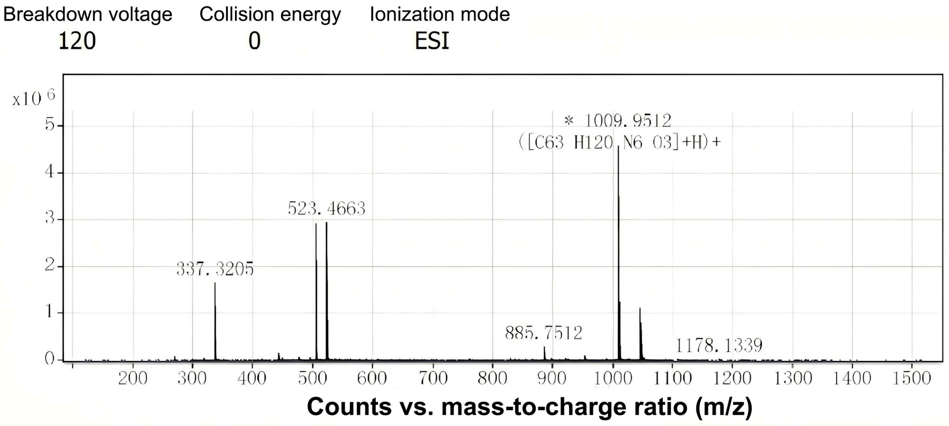


| *m/z* | z | abundance | molecular formula | ion |
|---|---|---|---|---|
| 337.3205 | 1 | 1652503.75 | | |
| 337.6549 | 1 | 1149090.75 | | |
| 505.4777 | 2 | 2908348 | | |
| 505.9792 | 2 | 2131276.5 | | |
| 523.4663 | 2 | 2939667 | | |
| 523.9676 | 2 | 2141779.25 | | |
| 524.4664 | 2 | 1699189.5 | | |
| 1009.9512 | 1 | 4571196 | C63 H120 N6 O3 | (M+H)+ |
| 1010.9531 | 1 | 3489256.75 | C63 H120 N6 O3 | (M+H)+ |
| 1011.9553 | 1 | 1232536 | C63 H120 N6 O3 | (M+H)+ |

**Figure S1.** Molecular structure identification of BXA2-8 ionizable lipid by HRMS.

.

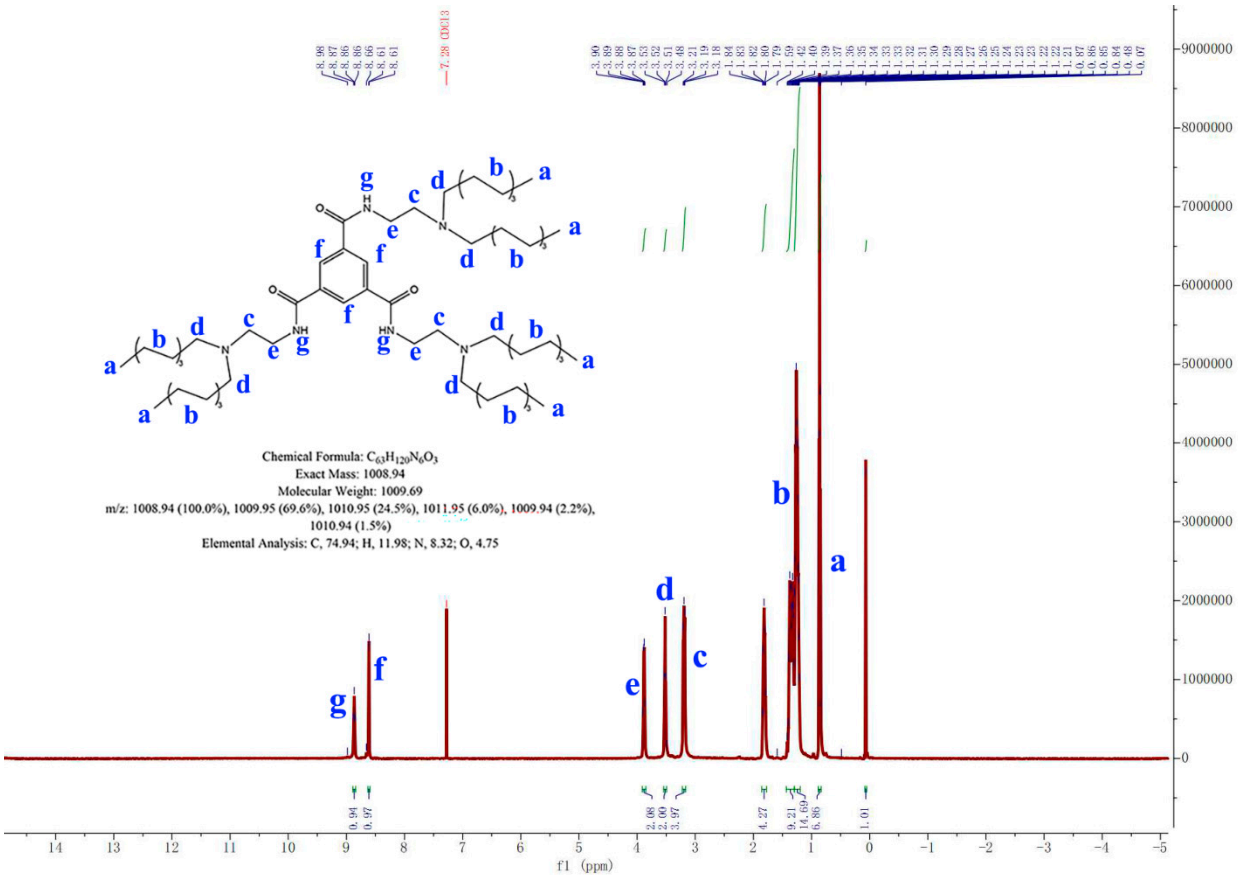


**Figure S2.** Molecular structure identification of BXA2-8 by $^{1}$H NMR.

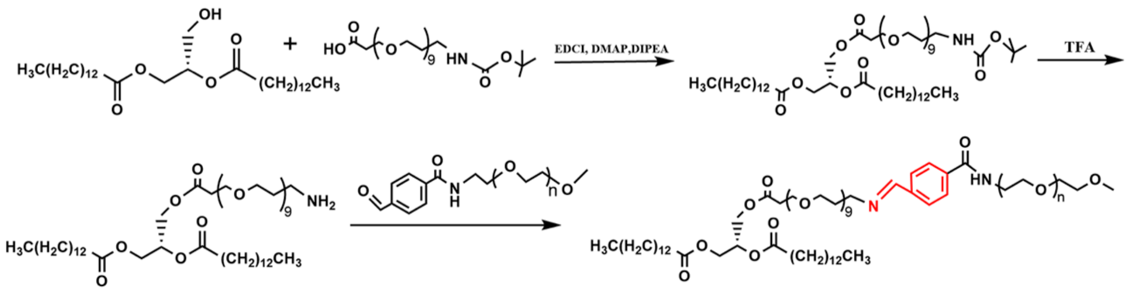


**Figure S3.** The synthetic route of Ben-mPEG2000.

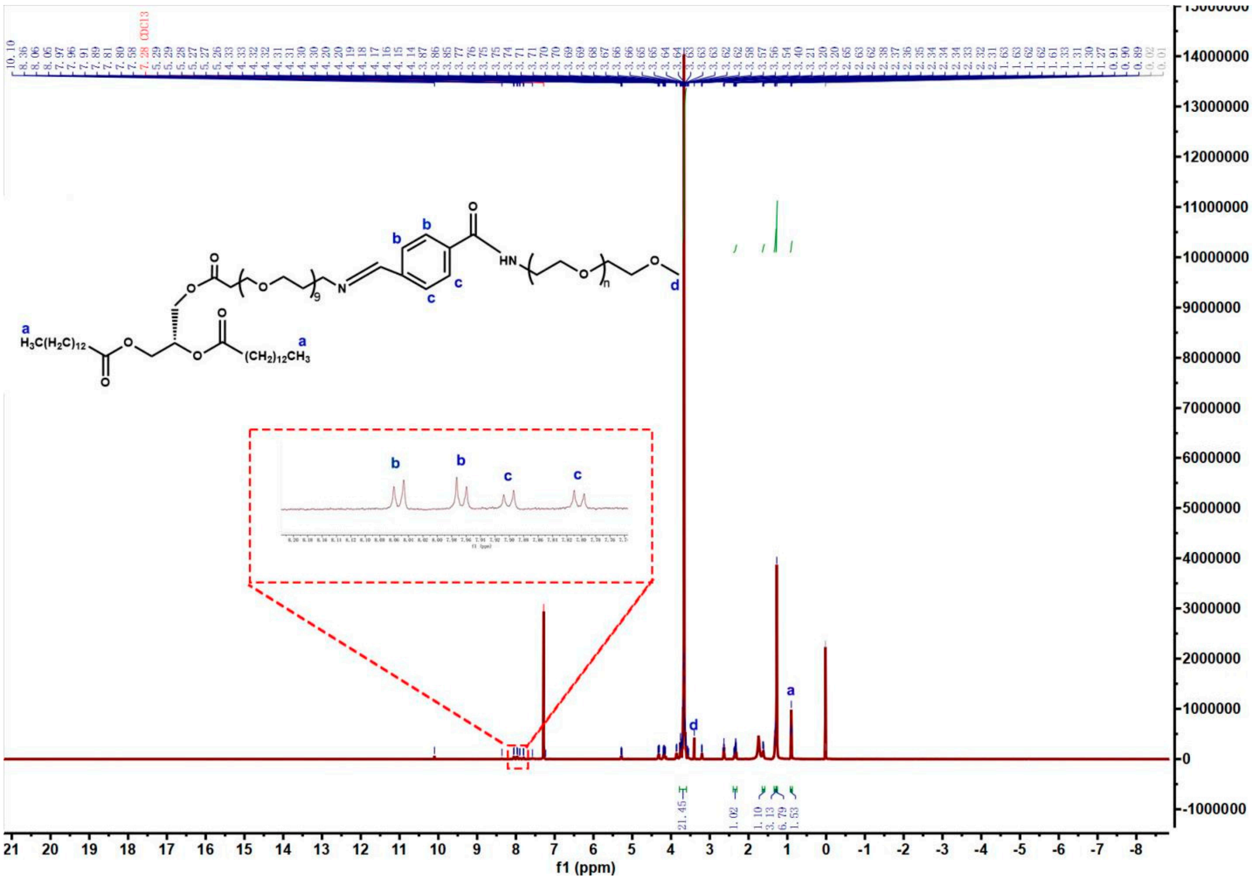


**Figure S4.** Molecular structure identification of Ben-mPEG2000 by $^{1}$H NMR.

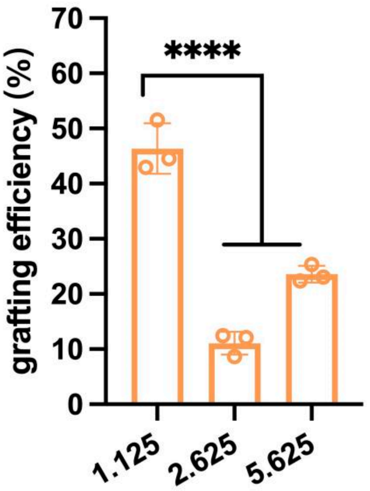


**Figure S5.** Grafting efficiency of Ben-mPEG2000 with different molar ratios (n=3). Significant differences were assessed using a one-way ANOVA with Tukey test ($^{****}p < 0.0001$). Data are presented as mean ± SD.

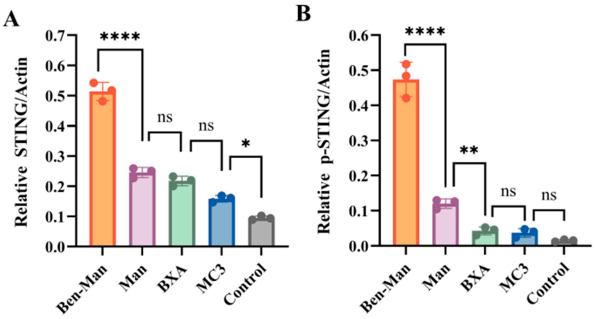


**Figure S6.** Quantitative analysis of (A) STING and (B) p-STING protein expression by Western blot in vitro. β-ACTIN was used as an internal control (n = 3). A one-way ANOVA with Tukey test was used to assess significant differences ($^{*}p < 0.05$; $^{**}p < 0.01$; $^{****}p < 0.0001$; ns: no significant difference). Data are presented as mean ± SD.

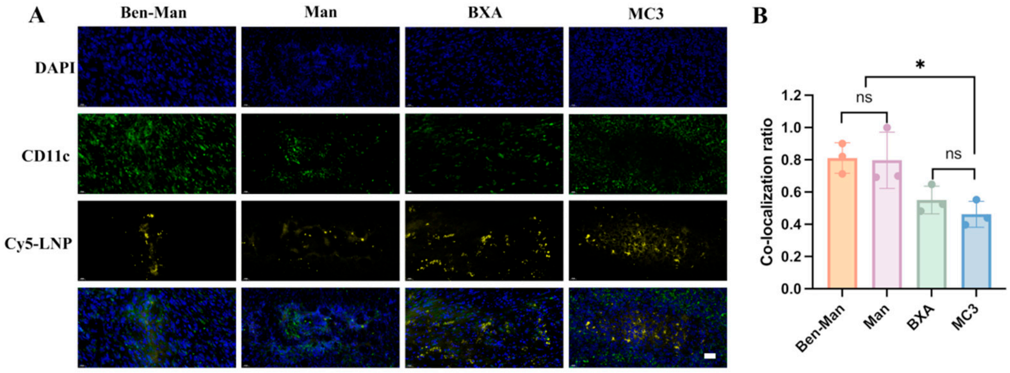


**Figure S7.** (A) The co-localization of Cy5-LNPs with DCs in lymph nodes was detected by the immunofluorescence technique, and (B) quantitative analysis was performed (scale = 200 μm, n = 3, x ± SD, $^{*}p < 0.05$, ns: no significant difference).

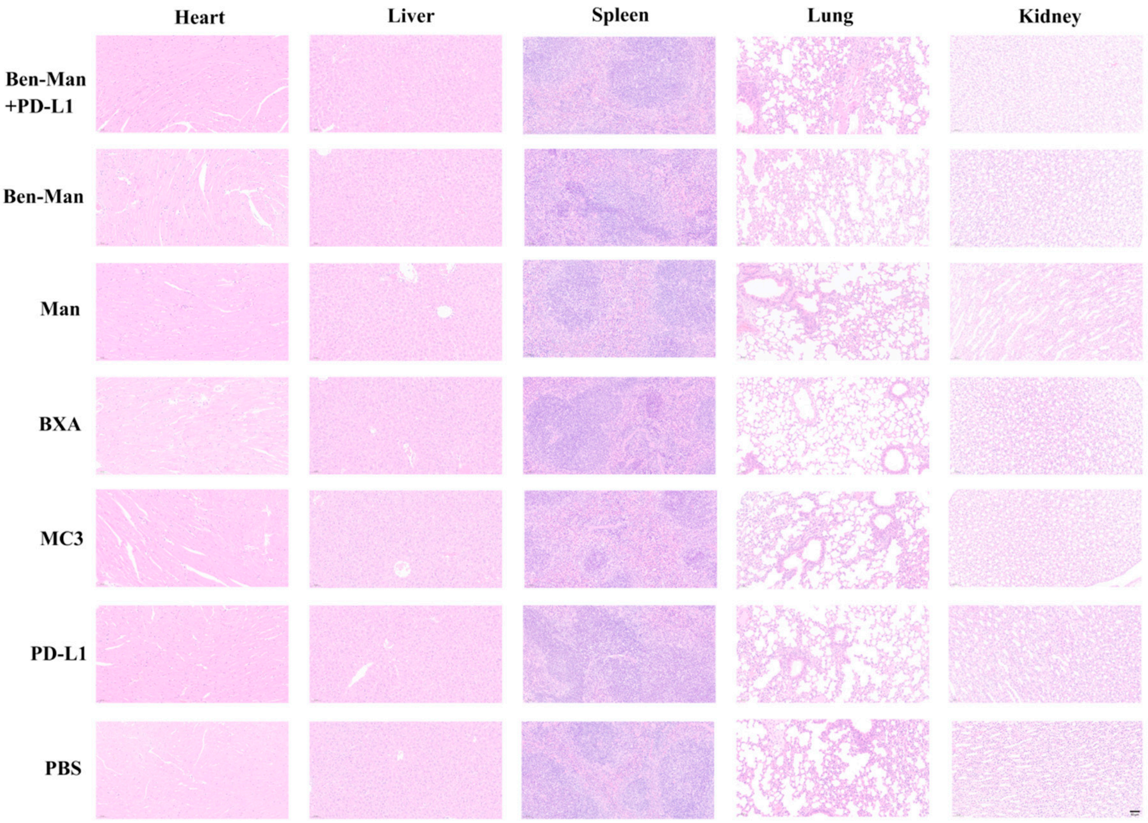


**Figure S8.** H&E staining analysis of important tissues (scale bar = 50 μm).

**Table S1.** The physicochemical properties of LNPs.

| Formulation | Size (nm) | PDI | EE (%) |
|---|---|---|---|
| Ben-Man | 125.57 ± 3.30 | 0.16 ± 0.004 | 96.49 ± 0.07 |

## Reference

1. Wang, S.; Zheng, J.; Zhou, J.; Jiang, W.; Chen, Z.; Wu, X.; Guo, B.; Wu, Y.; Yang, F. Engineered lipid nanoparticles with synergistic dendritic cell targeting and enhanced endosomal escape for boosted mRNA cancer vaccines. *Mater. Today Bio* **2025**, *34*, 102107.
